# SETDB2 Mitigates Podocyte Dysfunction in Diabetic Kidney Disease Through Epigenetic Silencing of SMAD3

**DOI:** 10.1002/advs.202516984

**Published:** 2025-11-29

**Authors:** Lanfang Li, Shimin Jiang, Qi Jin, Peng Qu, Yingjie Guo, Xushan Lan, Xinyu Li, Cuiting Sun, Sinan Ai, Xin Li, Weiliang Sun, Jing Guo, Wenge Li, Liang Peng, Lihong Liu

**Affiliations:** ^1^ Beijing Key Laboratory for Immune‐Mediated Inflammatory Diseases Institute of Clinical Medical Sciences China‐Japan Friendship Hospital Beijing 100029 China; ^2^ Department of Nephrology China‐Japan Friendship Hospital Beijing 100029 China; ^3^ Guang'anmen Hospital China Academy of Chinese Medical Sciences Beijing 100053 China; ^4^ China‐Japan Friendship Hospital Capital Medical University Beijing 100000 China; ^5^ Diabetes Department of integrated Chinese and Western medicine China‐Japan Friendship Hospital Beijing 100029 China; ^6^ China‐Japan Friendship Hospital (Institute of Clinical Medical Sciences) Chinese Academy of Medical Sciences & Peking Union Medical College Beijing 100730 China; ^7^ Department of Pharmacy China‐Japan Friendship Hospital Beijing 100029 China

**Keywords:** Diabetic kidney disease, epigenetic regulation, histone methylation, podocyte dysfunction, SETDB2, SMAD3

## Abstract

Podocyte dysfunction represents both an early pathological hallmark and a key driver of proteinuria in diabetic kidney disease (DKD); nevertheless, the potential epigenetic regulatory mechanisms remain poorly defined. Here, the histone methyltransferase SETDB2 is identified as a pivotal epigenetic suppressor of podocyte dysfunction and DKD progression. Glomerular SETDB2 expression exhibits a significant reduce in both DKD patients and mouse models, showing an inverse correlation with disease severity. Podocyte‐specific SETDB2 deficiency exacerbates podocytes dysfunction and accelerates DKD progression, whereas its overexpression exerts renal protective effects. Mechanistically, SETDB2 directly enhances H3K9 trimethylation at the *Smad3* promoter, thereby repressing SMAD3 expression and activation, ultimately preserving podocyte function. Notably, it identifies TCF21, a transcription factor downregulated in DKD, as a direct upstream regulator of *Setdb2* expression via binding to promoter and activating its transcription. Collectively, these findings establish SETDB2 as a critical regulator of podocyte integrity and a promising therapeutic target for DKD.

## Graphical abstract

SETDB2 epigenetically represses *Smad3* transcription by increasing H3K9me3 enrichment at its promoter, thereby mitigating podocyte dysfunction in DKD. The transcription factor TCF21 binds directly to the *Setdb2* promoter and enhances its expression in podocytes.

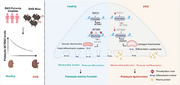

## 1 Introduction

Diabetic kidney disease (DKD) is a major contributor to end‐stage kidney disease and the cardiovascular events and mortality of diabetic populations, imposing a substantial global health and socioeconomic burden.^[^ ^1^ ^]^ Podocytes are essential for the glomerular filtration barrier but are highly vulnerable to metabolic and hemodynamic stress in diabetes.^[^ ^2^ ^]^ Hyperglycemia‐induced injury triggers cytoskeletal disorganization, loss of key podocyte markers, mesenchymal transition, and apoptosis, ultimately resulting in podocyte depletion and the progression from microalbuminuria to proteinuria in diabetic kidney disease.^[^ ^3^, ^4^, ^5^ ^]^ Therefore, elucidating the molecular mechanisms driving podocyte dysfunction is key to developing effective DKD therapies.^[^ ^6^ ^]^

Aberrant gene and protein expression in renal cells drives pathological changes and accelerates disease progression in diabetes.^[^ ^7^ ^]^ Such dysregulation arises not only from genetic variations but also from epigenetic mechanisms.^[^ ^8^ ^]^ Epigenetics involves heritable gene expression changes without DNA sequence variation, mediated mainly by DNA methylation, histone modifications, and non‐coding RNAs.^[^ ^9^ ^]^ Histone modifications‐including methylation, acetylation, phosphorylation, and ubiquitination‐could modulate chromatin structure and gene transcription in a context‐dependent manner.^[^ ^10^ ^]^ In diabetic settings, podocytes are exquisitely sensitive to epigenetic reprogramming under metabolic and inflammatory stress, and histone methylation has emerged as a central layer governing their fate decisions.^[^ ^11^ ^]^ Hyperglycemia‐induced epigenetic imbalance is characterized by an increase in activating marks (e.g., H3K4me3) and decreased repressive marks (e.g., H3K9me3, H3K27me3) at the promoters of pathogenic genes, thereby promoting podocyte dysfunction and disease progression.^[^ ^12^ ^]^ As is well known, histone methylation is dynamically regulated by opposing histone methyltransferases (“writers”) and demethylases (“erasers”), whose activities determine chromatin accessibility and transcriptional output.^[^ ^13^ ^]^ Currently, systematic investigations into the enzymes and signaling cascades that govern critical histone methylation sites in podocytes remain limited, thereby constraining the advancement of precision therapeutic strategies. The present study delineates the mechanistic basis of histone methylation‐mediated regulation in podocyte dysfunction, offering important insights into the epigenetic mechanisms underlying in DKD. SETDB2 (SET domain bifurcated 2) is a histone methyltransferase (HMT) belonging to the KMT1 family including SUV39H1, SUV39H2, SETDB1, G9a, and GLP‐that catalyze the trimethylation of histone H3 at lysine 9 sites (H3K9me3), which is a key epigenetic mark linked to transcriptional repression via chromatin compaction.^[^ ^14^ ^]^ Functionally, SETDB2 functions as a critical modulator of inflammatory and metabolic responses. In immune cells, it suppresses pro‐inflammatory gene transcription by depositing H3K9me3 at NF‐κB target sites. Its expression is inducible by interferon IFN‐β and critically governs the resolution of inflammatory responses and sustains immune homeostasis.^[^ ^15^ ^]^ In diabetic contexts, reduced SETDB2 levels have been observed in macrophages, contributing to persistent M1 polarization and impaired wound healing.^[^ ^16^ ^]^ Moreover, recent evidence indicates that SETDB2 regulates hepatic lipid metabolism involved in fatty acid synthesis and storage.^[^ ^17^ ^]^ Despite these advances, the role and regulatory mechanism of SETDB2 in the kidney, particularly in the context of DKD, has yet to be elucidated.

In this study, we identified *Setdb2* as one of the most significantly downregulated HMT genes in glomeruli from DKD mice, a finding that was further validated in human renal biopsies spanning multiple stages of disease severity. Glomerular SETDB2 expression exhibited a significant inverse correlation with DKD progression. Functional experiments revealed that podocyte‐specific SETDB2 deficiency exacerbated podocyte dysfunction and DKD progression, whereas its overexpression exerts a moderate protective effect. Mechanistically, integrative Cleavage Under Targets and Tagmentation (CUT&Tag) with transcriptomic analyses, combined with molecular validation, demonstrated that SETDB2 represses *Smad3* transcription through H3K9 trimethylation at its promoter, thereby attenuating SMAD3‐driven podocyte dysfunction. Collectively, our findings uncover an uncharacterized protective mechanism of SETDB2 in preserving podocyte integrity and highlight its therapeutic potential in DKD.

## 2 Results

### 2.1 Podocyte SETDB2 Expression is Reduced in Patients and Mice with DKD

**Figure 1:**
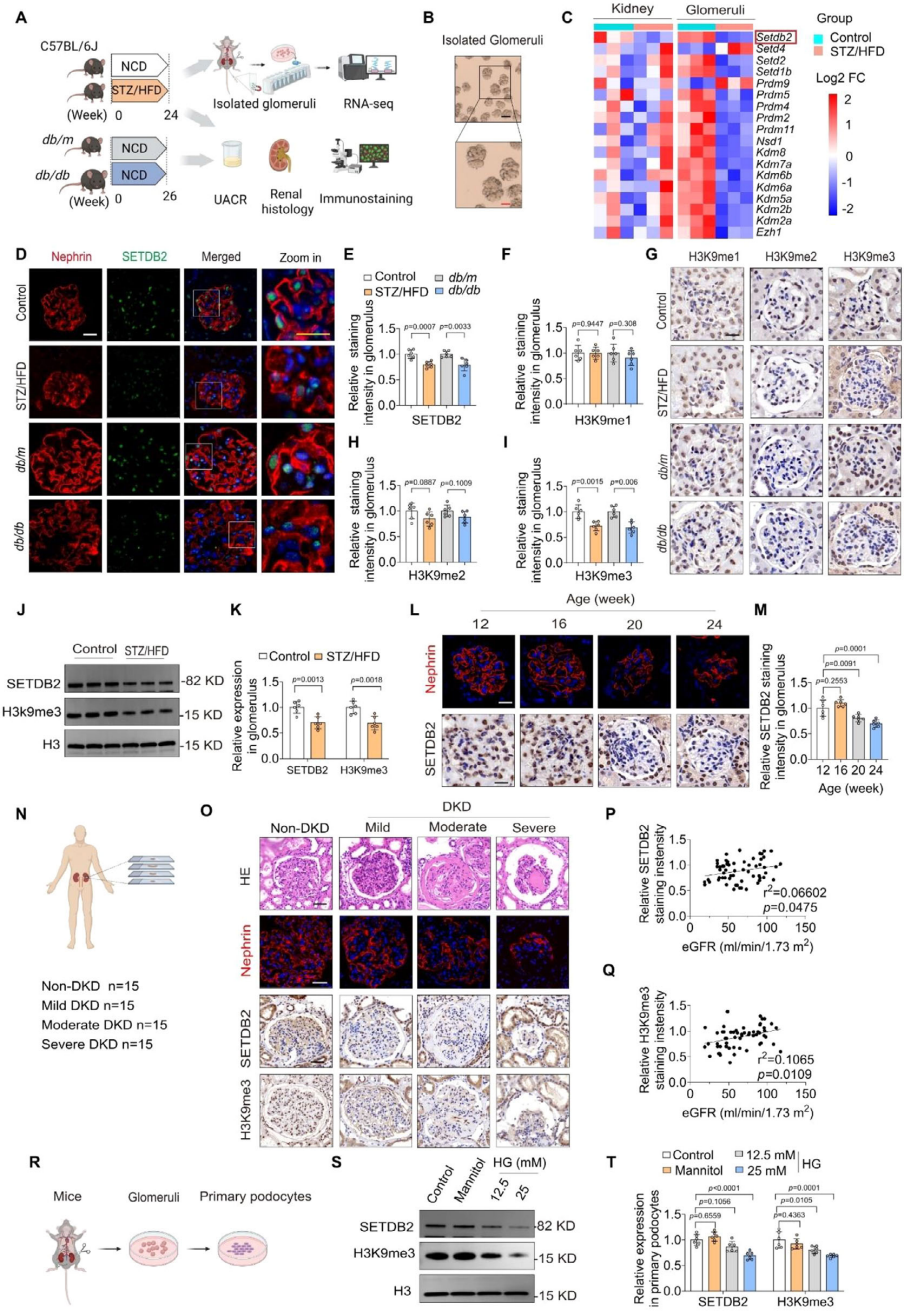
SETDB2 expression is reduced in patients and mice with DKD. A) Schematic diagram of experimental design in mice. B) Representative isolated glomeruli images imaged by light microscopy (purity> 98%). Scale bar, 100 µm (black), 50 µm (red). C) Heatmaps showing multiple genes related to histone methylation differentially expressed in kidney and glomeruli from control and STZ/HFD‐induced DKD mice. D,E) Representative immunofluorescent (IF) images of Nephrin (red) and SETDB2 (green) and quantitative analysis of SETDB2 expression in different experimental groups mice (n  = 6). Scale bar, 20 µm (white), 10 µm (yellow). F–I) Representative immunohistochemistry (IHC) images of H3K9me1, H3K9me2, and H3K9me3 expression and quantitative analysis in different experimental groups mice (n = 6). Scale bar, 20 µm. J,K) Representative Western blot images of SETDB2 and H3K9me3 expression and quantitative analysis in glomeruli from control and STZ/HFD‐induced DKD mice (n  = 6). L,M) Representative IF images of SETDB2 and quantitative analysis in different age STZ/HFD‐induced DKD mice from 12 to 24 week (n  = 6). Scale bar, 20 µm. N) Schematic diagram of experimental design in human samples. O) Representative images of HE staining, Nephrin (IF), SETDB2, and H3K9me3(IHC) images in human samples from non‐DKD controls (n = 15) and DKD patients (n = 45) with varying disease severity (mild to severe), categorized by estimated glomerular filtration rate (eGFR). Scale bar, 50 µm. P,Q) Correlation analysis between the expression of SETDB2 or H3K9me3 and eGFR (n = 60). R) Schematic diagram of the experimental design, from glomeruli isolation to primary podocytes culture. S,T) Representative Western blot images of SETDB2 and H3K9me3 expression and quantitative analysis in cultured primary podocytes with different concentrations of high glucose (HG) treatment (n  = 6). Data are presented as Mean±SD. Two‐tailed Student's *t*‐test analysis (E, F, H, I, K), Pearson's correlation analysis (P, Q), One‐way ANOVA with Tukey's post‐test (M, T), with *p* values indicated.

To characterize the transcriptional profile of HMTs in DKD, RNA sequencing was performed on whole kidneys and glomeruli from DKD mice (**Figure** **1**A,B; Figure S1A–E, Supporting Information). Among the differentially expressed HMT genes, *Setdb2* exhibited the pronounced downregulation, particularly within the glomerular compartment (Figure 1C; Figure S1F,G, Supporting Information). We further examined SETDB2 expression in two well‐established DKD mouse models: *db/db* mice and streptozotocin / high‐fat diet combined with unilateral nephrectomy (STZ/HFD)‐induced diabetic mice. In both models, glomerular SETDB2 expression was markedly decreased compared to controls, accompanied by decreased levels of its catalytic product H3K9me3, while the H3K9me1 or H3K9me2 levels remained unchanged (Figure 1D–I). These findings were also validated in glomeruli by Western blot analysis (Figure 1J,K; Figure S1H,I, Supporting Information). Notably, SETDB2 expression declined in a time‐dependent manner following the onset of proteinuria (Figure 1L,M).

To validate these findings in human samples, we assessed SETDB2 expression in renal biopsies from 45 DKD patients with varying disease severity (mild to severe), classified by estimated glomerular filtration rate (eGFR), alongside 15 non‐DKD controls samples derived from renal tissues adjacent to tumors in nephrectomized patients (Figure 1N). Immunostaining analysis revealed significantly reduced SETDB2 expression in glomeruli of DKD patients, which positively correlated with eGFR (Figure 1O,P S; Figure S1J,K, Supporting Information). Consistently, glomerular H3K9me3 levels paralleled SETDB2 expression (Figure 1O,Q), reinforcing potential role of the SETDB2‐H3K9me3 axis in DKD pathogenesis.

Double immunofluorescence staining of SETDB2 together with podocyte marks (Nephrin or WT1) further localized this reduction specifically to podocytes, rather than glomerular endothelial cells and mesangial cells (Figure 1D; Figure S2A, Supporting Information). In vitro, high‐glucose (HG) exposure reduced SETDB2 expression in primary podocytes and induced actin cytoskeleton disorganization (Figure 1R–T; Figure S2B–D, Supporting Information). By contrast, SETDB2 expression remained largely unchanged in glomerular endothelial cells (GECs) and mesangial cells (MSC) under similar conditions (Figure S2E–H, Supporting Information). Moreover, SETDB2 downregulation was also observed in adriamycin (ADR)‐induced nephropathy mice, a well‐established animal model of proteinuria and glomerulosclerosis caused by podocyte injury (Figure S2I–K, Supporting Information), highlighting the broader relevance of SETDB2 in maintaining podocyte integrity. Together, these findings indicated that the reduction of podocyte SETDB2 is a conserved feature across multiple models of glomerular injury and may contribute to DKD pathogenesis.

### 2.2 Podocyte‐Specific SETDB2 Deficiency Aggravates DKD Progression

**Figure 2:**
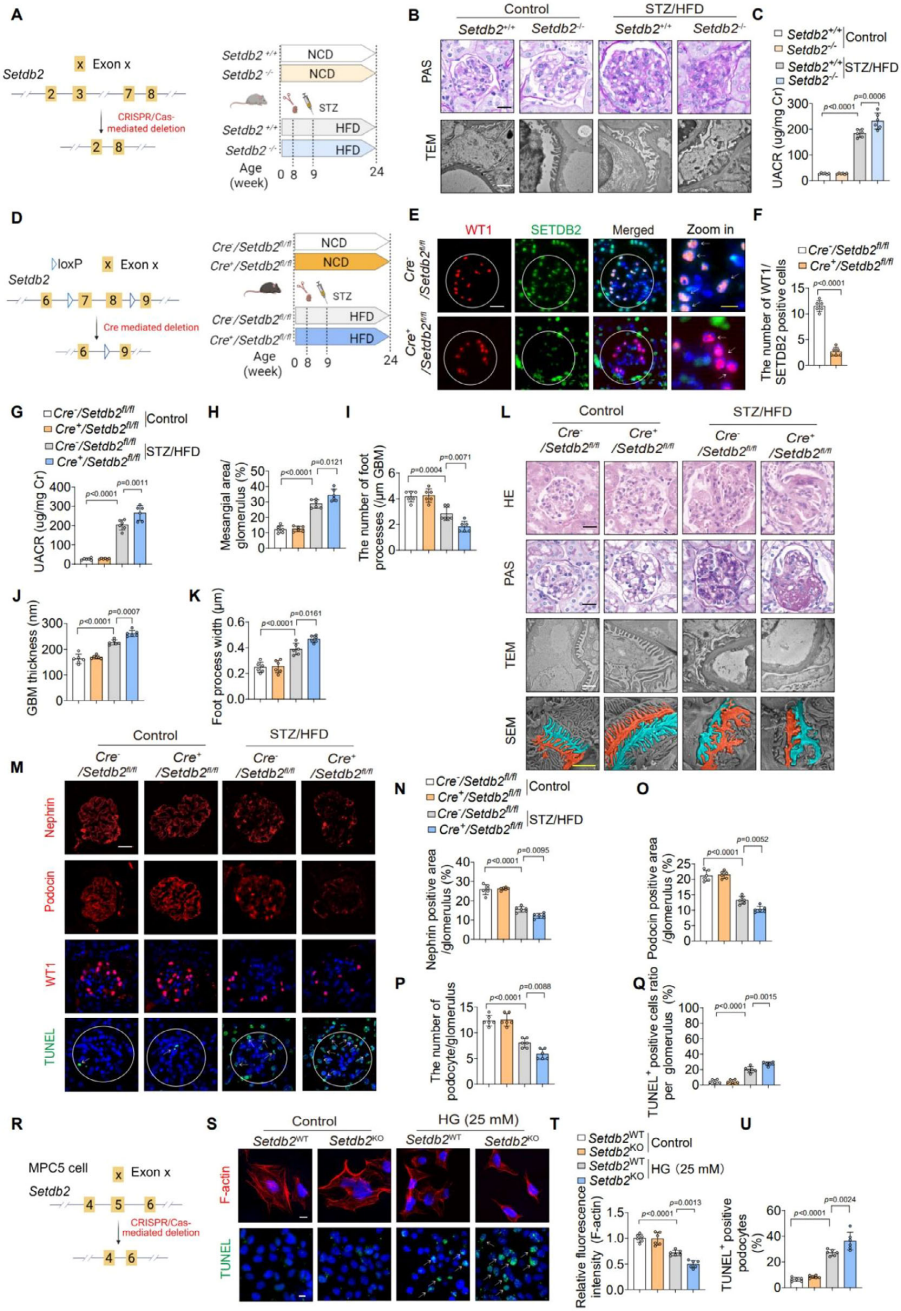
Podocyte‐specific SETDB2 deficiency aggravates DKD progression. A) Schematic diagram of homozygotes *Setdb2* knockout mice and the construction of the STZ/HFD‐induced DKD mice model. B) Glomerular structural changes were evaluated by PAS staining (n = 8) and Transmission Electron Microscopy (TEM) (n = 6) in *Setdb2^+/+^* and *Setdb2^−/−^* mice with or without STZ/HFD treatment. Scale bar, 20 µm (black), 1 µm (white). C) UACR in *Setdb2^+/+^* and *Setdb2^−/−^* mice with or without STZ/HFD treatment (n  = 6). D) Schematic diagram of design for producing the *Cre^+^*/*Setdb2^fl/fl^* mice and the construction of the STZ/HFD‐induced DKD model. E,F) Representative IF images showing the co‐localization analysis of Wilms' tumor 1 (WT‐1, a podocyte‐specific marker, red) and SETDB2 (green) in *Cre* ^−^/*Setdb2* ^fl/fl^ and *Cre* ^+^/*Setdb2* ^fl/fl^ mice (n  = 6). Scale bar, 20 µm (white), 10 µm (yellow). G) UACR in *Cre* ^−^/*Setdb2* ^fl/fl^ and *Cre* ^+^/*Setdb2* ^fl/fl^ mice with control or STZ/HFD treatment (n = 6). H–L) Representative images of HE, PAS, TEM, and Scanning Electron Microscope (SEM) staining and quantitative analysis in *Cre* ^−^/*Setdb2* ^fl/fl^ and *Cre* ^+^/*Setdb2* ^fl/fl^ mice with control or STZ/HFD treatment (n  = 6). Scale bar, 20 µm (black), 1 µm (white), 2 µm (yellow). M–Q) Representative IF images of Nephrin, Podocin, WT‐1, and TUNEL staining and quantitative analysis in *Cre* ^−^/*Setdb2* ^fl/fl^ and *Cre* ^+^/*Setdb2* ^fl/fl^ mice with control or STZ/HFD treatment (n  = 6). Scale bar, 20 µm. R) Schematic diagram of design for knockout the mouse *Setdb2* gene (*Setdb2* ^KO^) by CRISPR/Cas9 gene editing technique in the MPC5 cells. S–U) Representative IF images of F‐actin (staining by phalloidine, red, top) and TUNEL staining (green, bottom) and quantitative analysis in *Setdb2* ^KO^ with *Setdb2* ^WT^ MPC5 cells with or without HG (n = 6). Scale bar, 10 µm. Data are presented as Mean±SD. Two‐tailed Student's t‐test analysis (F), Two‐way ANOVA with Tukey's post‐test (C, G‐K, N‐Q, T‐U), with *p* values indicated.

To determine whether SETDB2 loss contributes causally to DKD rather than representing a secondary consequence, we first generated homozygous *Setdb2* knockout (*Setdb2* ^−/−^) mice and subjected them to STZ/HFD‐induced diabetic stress (**Figure** **2**A; Figure S3A–E, Supporting Information). Compared with wild‐type controls, SETDB2‐deficient mice exhibited more severe glomerular pathology, characterized by GBM thickening, podocyte foot process effacement, mesangial matrix expansion, podocyte‐specific markers loss (Nephrin, Podocin, and WT1), and increased podocyte apoptosis (Figure 2B,C; Figure S3F‐N, Supporting Information). These findings support a pathogenic role for SETDB2 deficiency in DKD.

To further elucidate the podocyte‐specific function of SETDB2 in DKD, we produced podocyte‐specific SETDB2‐deficient mice by hybridizing *Setdb2* ^flox/flox^ mice (*Setdb2* ^fl/fl^) with *Nphs2*‐*Cre* mice (Figure 2D). Efficient deletion was validated by tail genotyping and Western blot analysis of primary podocytes (Figure S4A–C, Supporting Information). Immunofluorescence analysis further verified the loss of SETDB2 specifically in podocytes of *Cre* ^+^/*Setdb2* ^fl/fl^ mice (Figure 2E,F), accompanied by the reduction in H3K9me3 levels as shown by immunohistochemistry (IHC) (Figure S4D–F, Supporting Information). Both *Cre* ^−^/*Setdb2* ^fl/fl^ and *Cre* ^+^/*Setdb2* ^fl/fl^ mice were subjected to STZ/HFD‐induced DKD. Podocyte‐specific SETDB2‐deficient mice exhibited worsened renal dysfunction, as verified by elevated urinary albumin‐to‐creatinine ratio (UACR) (Figure 2G). Histological examination revealed exacerbated mesangial matrix expansion and ultrastructural abnormalities, including GBM thickening and podocyte foot process effacement, in SETDB2‐deficient mice (Figure 2H–L, Supporting Information). Consistently, the expression of podocyte‐specific markers (Nephrin, Podocin, and WT1) was further reduced, and podocyte apoptosis was increased in *Cre* ^+^/*Setdb2* ^fl/fl^ mice (Figure 2M–Q, Supporting Information), indicating that SETDB2 deficiency promotes podocyte loss. In vivo, F‐actin staining demonstrated reduced filamentous actin, while TUNEL assays confirmed enhanced podocytes apoptosis in SETDB2‐deficient MPC5 cells (Figure 2R–U; Figure S4G–I, Supporting Information). Together, these findings demonstrate that podocyte‐specific SETDB2 deficiency accelerates DKD progression.

### 2.3 Podocyte‐Specific SETDB2 Deficiency Promotes Podocyte Dysfunction in DKD Mice

**Figure 3:**
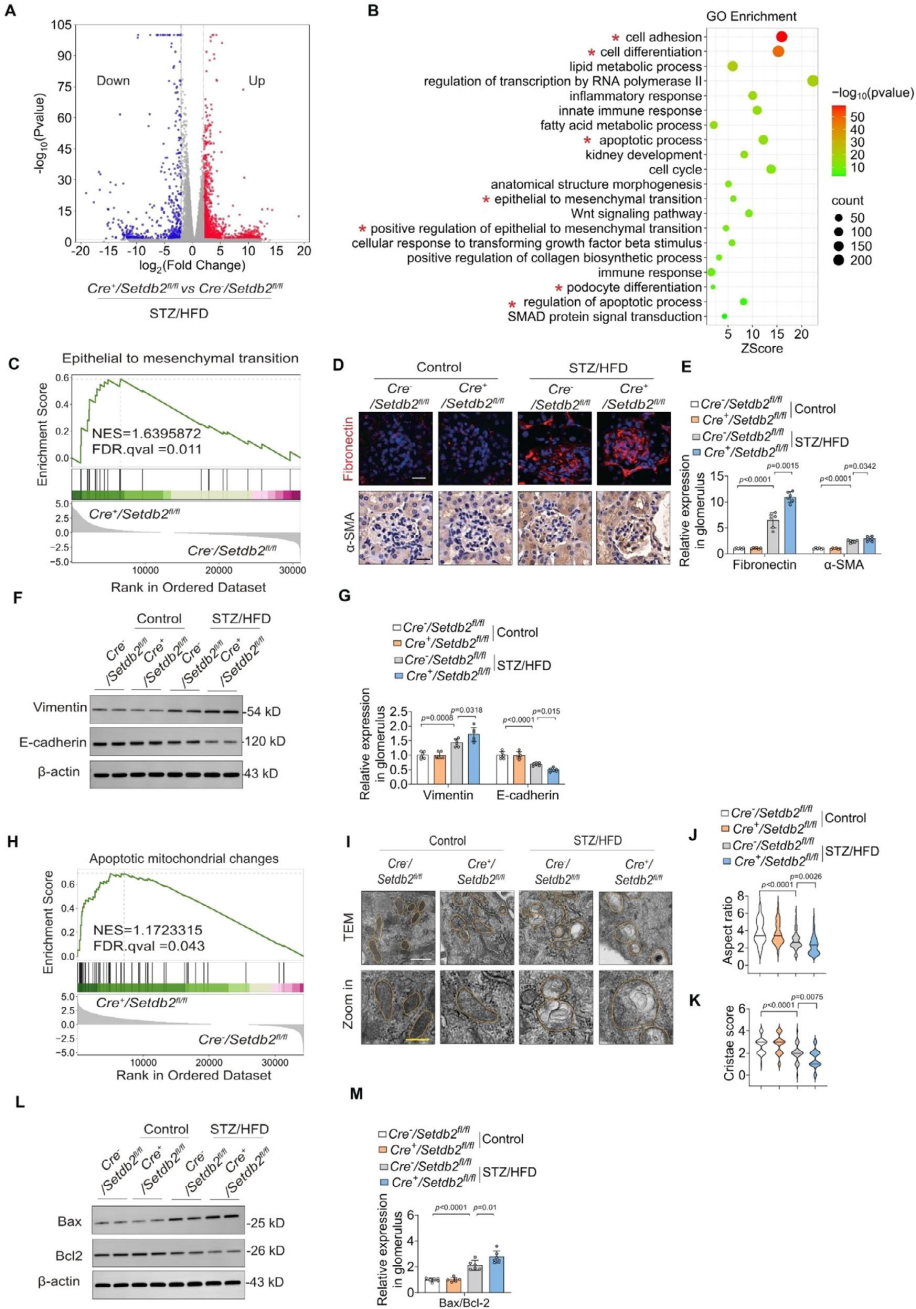
Podocyte‐specific SETDB2 deficiency promotes podocyte dysfunction in DKD mice. A) A Volcano plot showing the genes expression changes in the glomeruli from *Cre* ^+^/*Setdb2* ^fl/fl^ and *Cre* ^−^/*Setdb2* ^fl/fl^ mice with STZ/HFD treatment (red, upregulated genes; blue, downregulated genes, Log2FC >2, *p*< 0.05). B) GO enrichment analysis of the differential expression genes (DEGs) in the glomeruli from the *Cre* ^−^/*Setdb2* ^fl/fl^ and *Cre* ^+^/*Setdb2* ^fl/fl^ mice with STZ/HFD treatment. C) GSEA showed activation of genes related to the epithelial‐mesenchymal transition (EMT) pathways in *Cre* ^+^/*Setdb2* ^fl/fl^ mice with STZ/HFD treatment, compared to the *Cre* ^−^/*Setdb2* ^fl/fl^ mice. D,E) Representative images of EMT molecular markers – Fibronectin (IHC) and α‐SMA (IF) and quantitative analysis in *Cre* ^−^/*Setdb2* ^fl/fl^ and *Cre* ^+^/*Setdb2* ^fl/fl^ mice with control and STZ/HFD treatment (n = 6). Scale bar, 20 µm. F,G) Representative Western blot images of EMT molecular markers (Vimentin and E‐cadherin) and quantitative analysis in glomeruli from *Cre* ^−^/*Setdb2* ^fl/fl^ and *Cre* ^+^/*Setdb2* ^fl/fl^ mice with control and STZ/HFD treatment (n = 6). H) GSEA of apoptotic mitochondrial changes pathways were significantly enriched in *Cre* ^+^/*Setdb2* ^fl/fl^ mice with STZ/HFD treatment, compared to the *Cre* ^−^/*Setdb2* ^fl/fl^ mice. I) Representative TEM images analyses of mitochondrion ultrastructure in podocyte from *Cre* ^−^/*Setdb2* ^fl/fl^ and *Cre* ^+^/*Setdb2* ^fl/fl^ mice with control and STZ/HFD treatment (n = 6). Scale bar: 500 nm (white), 250 nm (yellow). J) Quantitative analyses of mitochondrial aspect ratio using TEM micrographs of primary podocytes isolated from 6 mice/group (n  =  114 mitochondria (*Cre* ^−^/*Setdb2* ^fl/fl^ mice with control treatment), n  =  125 mitochondria (*Cre* ^+^/*Setdb2* ^fl/fl^ mice with control treatment), n  =  134 mitochondria (*Cre* ^−^/*Setdb2* ^fl/fl^ mice with STZ/HFD treatment) and n  =  123 mitochondria (*Cre* ^+^/*Setdb2* ^fl/fl^ mice with STZ/HFD treatment)). K) Quantification of mitochondrial cristae integrity using TEM micrographs of primary podocytes isolated from 6 mice/group (n  =  60 mitochondria). Mitochondrial cristae were scored using a semi‐quantitative 0–4 scale: 0, no cristae; 1, sparse residual cristae (<25% of normal); 2, partially preserved cristae (25–50%); 3, largely preserved cristae (50–75%); and 4, intact cristae (>75%). L,M) Representative Western blot images of Bax/Bcl2 and quantitative analysis in glomeruli from *Cre* ^−^/*Setdb2* ^fl/fl^ and *Cre* ^+^/*Setdb2* ^fl/fl^ mice with control and STZ/HFD treatment (n = 6). Data are presented as Mean±SD. Two‐way ANOVA with Tukey's pos*t*‐test (E, G, J, K, M), with *p* values indicated.

To investigate the molecular mechanisms by which podocyte SETDB2 loss exacerbates DKD progression, we performed RNA sequencing on glomeruli from *Cre* ^+^/*Setdb2* ^fl/fl^ and *Cre* ^−^/*Setdb2* ^fl/fl^ mice. A total of 2149 differentially expressed genes (DEGs) were identified, including 527 down‐regulated and 1622 up‐regulated genes (Log2FC> 2, *p* < 0.05) (**Figure** **3**A). Gene Ontology (GO) analysis revealed significant enrichment of these DEGs in various biological processes related to podocyte differentiation, epithelial‐to‐mesenchymal transition (EMT), immune responses, and apoptosis, all of which are implicated in DKD pathogenesis (Figure 3B).

During DKD progression, podocytes undergo dedifferentiation, characterized by the loss of podocyte‐and epithelial‐specific markers such as Nephrin, Podocin, and E‐cadherin, alongside the upregulation of mesenchymal markers including vimentin, fibronectin, and α‐SMA.^[^ ^4^ ^]^ Gene Set Enrichment Analysis (GSEA) revealed that genes markedly upregulated in *Cre* ^+^/*Setdb2* ^fl/fl^ mice were significantly enriched in the EMT pathway (Figure 3C). To determine whether SETDB2 deficiency contributes to these maladaptive changes, we analyzed protein expression in diabetic glomeruli. Immunostaining demonstrated upregulation of Fibronectin and α‐SMA in the glomeruli from STZ/HFD‐induced DKD mice, and the deposition of collagen is more pronounced; these alterations were further exacerbated in podocyte SETDB2‐deficient mice (Figure 3D,E; Figure S5A,B, Supporting Information). Consistently, the decrease expression of E‐cadherin, along with the increase levels of vimentin in diabetic mice were verified by Western blot analysis (Figure 3F,G), supporting the notion that SETDB2 deficiency promotes podocyte dedifferentiation and mesenchymal transition.

Among various mechanisms implicated in DKD, mitochondrial dysfunction has gained increasing attention.^[^ ^18^ ^]^ Diabetic podocytes display a spectrum of mitochondrial abnormalities, including elevated disrupted bioenergetics, and altered mitochondrial dynamics.^[^ ^19^ ^]^ These mitochondrial defects compromise cellular homeostasis and can trigger intrinsic apoptotic signaling, thereby exacerbating podocyte injury and loss.^[^ ^20^ ^]^ Our GSEA revealed that the DEGs in SETDB2‐deficient mice were significantly enriched in pathways related to mitochondrial dysfunction, including apoptosis and mitochondrial ATP synthesis (Figure 3H; Figure S5C, Supporting Information). Mitochondrial ultrastructural abnormalities, including swelling, reduced matrix density, cristae rupture, and membrane ablation, were detected in primary podocytes from STZ/HFD‐induced mice and were further exacerbated by podocyte‐specific *Setdb2* deletion (Figure 3I–K, Supporting Information). Western blot analysis also confirmed increased podocyte apoptosis in *Cre* ^+^/*Setdb2* ^fl/fl^ mice (Figure 3L,M). In vitro, *Setdb2* deletion significantly decreased ATP production and maximal respiratory capacity in podocytes with HG treatment (Figure S5D–H, Supporting Information), further demonstrating that SETDB2 deficiency impairs mitochondrial function. Together, these findings demonstrate that podocyte‐specific SETDB2 deficiency drives DKD progression by promoting podocyte dedifferentiation, EMT, and mitochondrial dysfunction.

### 2.4 SETDB2 Deficiency Promotes Podocytes Dysfunction via the Loss of H3K9me3‐Dependent Inhibition of SMAD3

**Figure 4:**
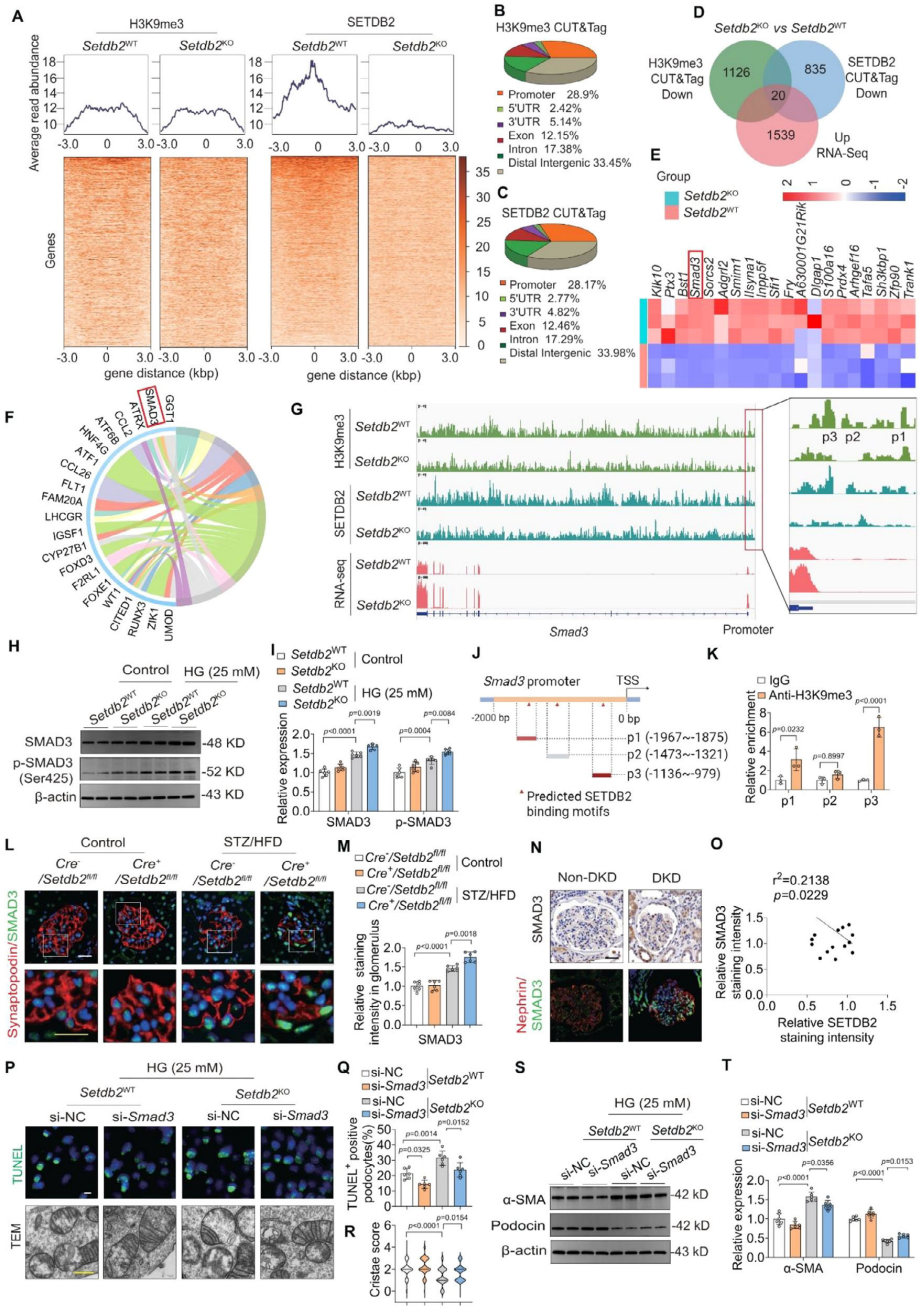
SETDB2 deficiency promotes podocytes dysfunction via H3K9me3‐dependent activation of SMAD3. A) Heatmaps of H3K9me3 signal (left) and SETDB2 signal (right) at TSS (± 3 kb) in *Setdb2* ^KO^ versus *Setdb2* ^WT^ MPC5 cells with HG treatment. B,C) Pie chart revealing the distribution of H3K9me3 and SETDB2 peaks on the genome comparing *Setdb2* ^KO^ with *Setdb2* ^WT^ MPC5 cells treated with HG. D) Venn chart revealing overlap of upregulated genes from RNA‐Seq and downregulated signal of H3K9me3 and SETDB2 mark at gene promoter regions in *Setdb2* ^KO^ with *Setdb2* ^WT^ MPC5 cells treated with HG. E) RNA‐Seq was performed in *Setdb2* ^KO^ with *Setdb2* ^WT^ MPC5 cells treated with HG. F) Chord diagram displaying functional enrichment relationships between differentially SETDB2 mark at promoter regions and enriched terms in *Setdb2* ^KO^ with *Setdb2* ^WT^ MPC5 cells with HG. G) Integrative genomics viewer (IGV) analysis representing H3K9me3 and SETDB2 peaks at the *Smad3* loci in *Setdb2* ^KO^ and *Setdb2* ^WT^ MPC5 cells treated with HG. H,I) Representative Western blot images of SMAD3 and p‐SMAD3 and quantitative analysis in *Setdb2* ^KO^ and *Setdb2* ^WT^ MPC5 cells with or without HG (n = 6). J) The schematic indicated the potential SETDB2‐H3K9me3 binding site in CUT&Tag assay. K) Relative enrichment of H3K9me3 on the specified regions of *Smad3* promoter in MPC5 cells detected by ChIP assay using anti‐H3K9me3 or control IgG antibody, analyzed by qPCR (n = 3). L,M) Representative fluorescence microscopic images revealing the expression of SETDB2 (green) and Synaptopodin (red) in *Cre* ^−^/*Setdb2* ^fl/fl^ and *Cre* ^+^/*Setdb2* ^fl/fl^ mice with control or STZ/HFD treatment (n  = 6). Scale bar, 20 µm (white), 10 µm (yellow). N) Representative images of Nephrin/SMAD3 (IF) and SMAD3 (IHC) in human renal tissues from non‐DKD controls and moderate DKD patients (n = 6). Scale bar, 50 µm. O) Correlation analysis between glomerular SETDB2 and SMAD3 expression in human samples from non‐DKD controls and DKD patients with varying disease severity (mild to severe) (n = 24). P) Representative TUNEL staining (IF) images and mitochondrial morphological changes in *Setdb2* ^KO^ and *Setdb2* ^WT^ MPC5 cells with si‐NC or si‐*Smad3* treatment (n = 6). Scale bar, 10 µm (white), 500 nm (yellow). Q) The quantitative analysis of TUNEL staining in *Setdb2* ^KO^ and *Setdb2* ^WT^ MPC5 cells with si‐NC or si‐*Smad3* intervention (n = 6). R) Quantification of mitochondrial cristae integrity using TEM micrographs of MPC5 cells (n  =  60 mitochondria (*Setdb2* ^WT^ MPC5 cells with si‐NC treatment), n  =  72 mitochondria (*Setdb2* ^WT^ MPC5 cells with si‐*Smad3* treatment), n  =  73 mitochondria (*Setdb2* ^KO^ MPC5 cells with si‐NC treatment) and n  =  73 mitochondria (*Setdb2* ^KO^ MPC5 cells with si‐*Smad3* treatment)). S,T) Representative Western blot images of α‐SMA and Podocin and quantitative analysis in *Setdb2* ^KO^ and *Setdb2* ^WT^ MPC5 cells with or without HG (n = 6). Data are presented as Mean±SD. Two‐tailed Student's *t*‐test analysis (K), Two‐way ANOVA with Tukey's post‐test (I, M, Q, R‐T), with *p* values indicated.

SETDB2 is known to repress gene transcription through H3K9me3‐mediated chromatin compaction. We hypothesized that SETDB2 deficiency increases chromatin accessibility by reducing H3K9me3, thereby promoting aberrant gene activation. To unmask the regulatory mechanism of SETDB2 in podocyte dysfunction, CUT&Tag was performed with H3K9me3 and SETDB2 antibody in *Setdb2*‐knockout (*Setdb2* ^KO^) and *Setdb2* wild‐type (*Setdb2* ^WT^) MPC5 cells (Figure S6A, Supporting Information). Both H3K9me3 and SETDB2 were enriched at gene promoter regions (**Figure** **4**A). In *Setdb2* ^KO^ cells, global H3K9me3 and SETDB2 binding peaks were significantly reduced, with ≈30% of these differential peaks localized to promoter regions (Figure 4B,C). By integrating CUT&Tag and RNA‐seq data, we identified 20 genes whose altered expression was directly associated with SETDB2‐mediated H3K9me3 marking (Figure 4D,E). Functional annotation revealed significant enrichment in pathways including cell differentiation, cell adhesion, cytoskeleton organization, etc. (Figure S6B, Supporting Information).

Among these, SMAD3 emerged as a key candidate due to prominent SETDB2/H3K9me3 co‐occupancy at its promoter and its significant upregulation upon *Setdb2* knockout (Figure 4F,G). We validated these findings by Western blot and q‐PCR analysis, which showed increased SMAD3 and phosphorylated SMAD3 (p‐SMAD3) protein levels and *Smad3* mRNA level in *Setdb2* ^KO^ MPC5 cells (Figure 4H,I; Figure S6C, Supporting Information). Concordantly, SETDB2 overexpression suppressed SMAD3 protein levels (Figure S6D,E, Supporting Information). To directly test whether SETDB2 modulates *Smad3* through H3K9me3, we performed H3K9me3 ChIP‐qPCR assays in SETDB2‐overexpressing MPC5 cells. This confirmed enrichment of H3K9me3 at the *Smad3* promoter (−1967–−1875 bp and −1136–−979 bp) (Figure 4J,K, Supporting Information).

Immunofluorescence staining in DKD mice showed similar SMAD3 elevation, which was further amplified by podocyte‐specific SETDB2 deficiency (Figure 4L,M, Supporting Information). In human DKD renal biopsies, SMAD3 levels were significantly elevated compared to non‐DKD controls and negatively correlated with the eGFR (Figure 4N; Figure S6J, Supporting Information). Importantly, SMAD3 expression inversely correlated with SETDB2 levels in glomeruli from DKD patients (Figure 4O). Functionally, silencing *Smad3* ameliorated apoptosis, mitochondrial abnormalities and filamentous actin loss in MPC5 cells (Figure 4P–R; Figure S6K,L, Supporting Information). In addition, silencing *Smad3* restored the expression of Podocin and reduced the abnormal increase of α‐SMA in MPC5 cells (Figure 4S,T).

SMAD3 also undergoes several post‐translational modifications, such as ubiquitination and phosphorylation, which may influence its expression and activation. However, our study revealed that the loss of SETDB2 did not significantly affect the expression of major regulators involved in SMAD3 post‐translational modifications in the presence of HG, including E3 ubiquitin ligases (NEDD4L, etc.)^[^ ^21^ ^]^ and phosphorylase (TβR1, DUSP1, etc.)^[^ ^22^, ^23^ ^]^ (Figure S6F,G, Supporting Information). TGFβ‐SMAD3 signaling is initiated by TGF‐β ligand binding to the TβRII/TβRI complex, which subsequently phosphorylates SMAD3.^[^ ^22^ ^]^ However, SETDB2 deficiency notably increased TGF‐β1 secretion from podocytes, the key upstream activator of SMAD3 (Figure S6H,I, Supporting Information).

Taken together, these results demonstrate that SETDB2 epigenetically suppress *Smad3* transcription and activation via H3K9me3 deposition at its promoter, whereas SMAD3 activation may also be associated with elevated TGF‐β1 levels, thereby mitigating podocyte dysfunction in DKD.

### 2.5 Podocyte‐Specific SETDB2 Overexpression Alleviates Podocyte Dysfunction in DKD

**Figure 5:**
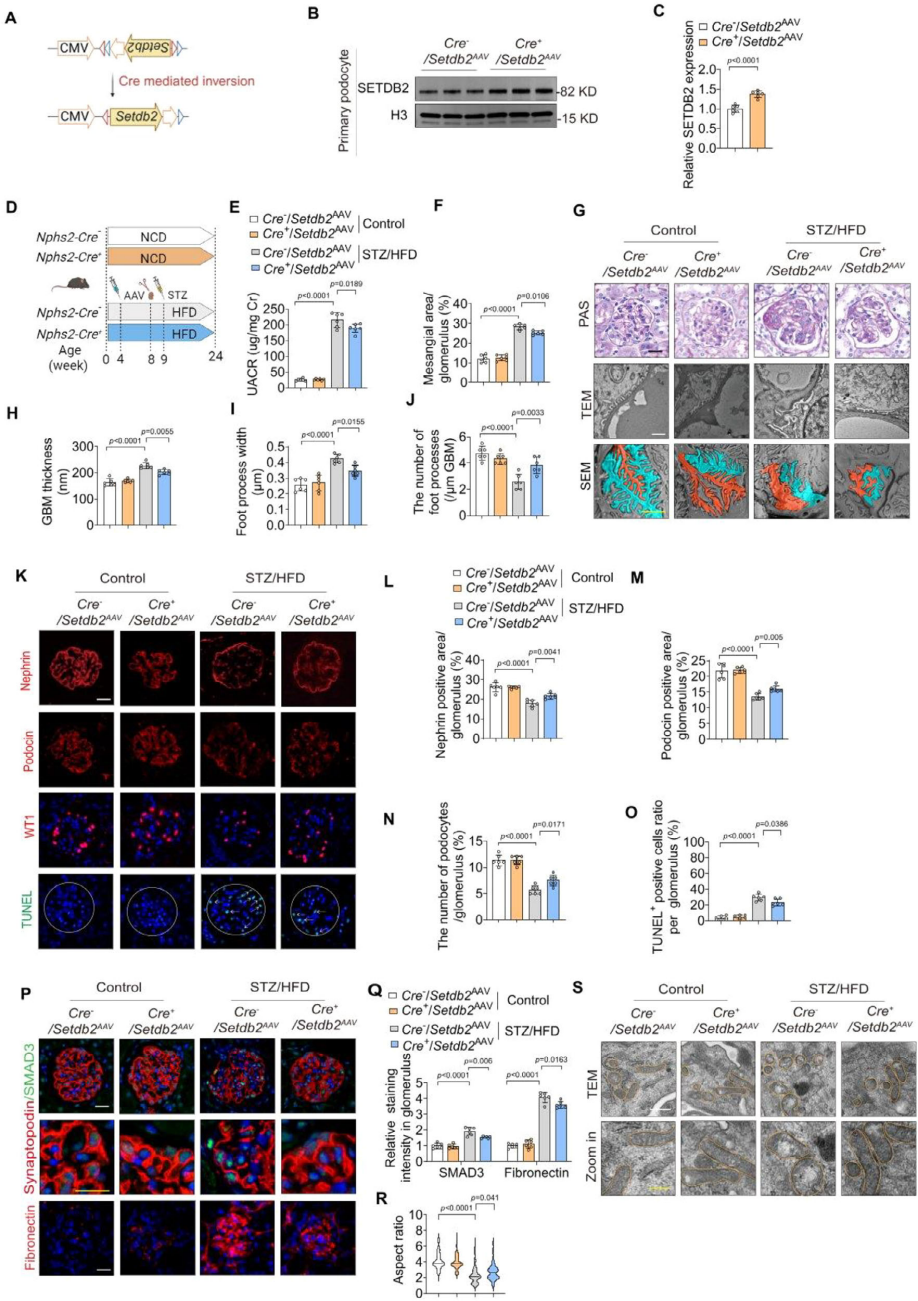
Podocyte‐specific SETDB2 overexpression alleviates podocyte dysfunction in DKD. A) Schematic design of the pHBAAV‐CMV‐DIO‐MCS used to generate podocyte‐specific overexpression SETDB2 mice. The construct was designed so that Cre induction could be used to orchestrates the transcriptional reconfiguration of *Setdb2* to sense orientation. B,C) Representative Western blot images of SETDB2 expression and quantitative analysis in primary podocytes from *Cre* ^−^/*Setdb2* ^AAV^ and *Cre* ^+^/*Setdb2* ^AAV^ mice. D) Experimental scheme for producing the STZ/HFD‐induced diabetic model in *Cre* ^−^/*Setdb2* ^AAV^ and *Cre* ^+^/*Setdb2* ^AAV^ mice. E) UACR in different experimental mouse groups (n = 6). F–J) Representative images of PAS, TEM, and SEM staining and quantitative analysis in different experimental mouse groups (n  = 6). Scale bar, 20 µm (black), 1 µm(red), 2 µm (yellow). K–O) Representative IF images of Nephrin, Podocin, WT‐1 and TUNEL and quantitative analysis in different experimental mouse groups (n  = 6). Scale bar, 20 µm. P,Q) Representative IF images of SMAD3/Synaptopodin and Fibronectin and quantitative analysis in different experimental mouse groups (n = 6). Scale bar, 20 µm (white), 10 µm (yellow). S) Quantitative analyses of mitochondrial aspect ratio using TEM micrographs of primary podocytes isolated from 6 mice/group (n  =  125 mitochondria (*Cre* ^−^/*Setdb2* ^AAV^ mice with control treatment), n  =  125 mitochondria (*Cre* ^+^/*Setdb2* ^AAV^ mice with control treatment), n  =  114 mitochondria (*Cre* ^−^/*Setdb2* ^AAV^ mice with STZ/HFD treatment) and n  =  116 mitochondria (*Cre* ^+^/*Setdb2* ^AAV^ mice with STZ/HFD treatment)). R) Representative TEM images analyses of mitochondrion ultrastructure in podocyte from *Cre* ^−^/*Setdb2* ^AAV^ and *Cre* ^+^/*Setdb2* ^AAV^ mice with control and STZ/HFD treatment (n = 6). Scale bar: 500 nm (white), 250 nm (yellow). Data are presented as Mean±SD. Two‐tailed Student's t‐test analysis (C), Two‐way ANOVA with Tukey's post‐test (E‐F, H‐J, L‐O, Q‐R), with *p* values indicated.

To evaluate the therapeutic potential of SETDB2 restoration in vivo, we delivered an AAV9 vector carrying a *Cre*‐dependent *Setdb2* overexpression construct into *Nphs2*‐*Cre* mice, which orchestrates the transcriptional reconfiguration of *Setdb2* to sense orientation, to produce podocyte‐specific *Setdb2* overexpression mice (*Cre* ^+^/*Setdb2* ^AAV^) (**Figure** **5**A). Robust SETDB2 overexpression in podocytes was verified by Western blot analysis, immunofluorescence staining, and qPCR analysis (Figure 5B,C; Figure S7A–D, S7). Following STZ/HFD challenge, *Cre* ^+^/*Setdb2* ^AAV^ mice exhibited significant renal protection compared to diabetic *Cre* ^−^/*Setdb2* ^AAV^ mice, as demonstrated by lower UACR, reduced mesangial expansion, normalized foot process width, and attenuated GBM thickening (Figure 5D–J). Immunostaining of podocyte marker showed that SETDB2 overexpression preserved podocyte identity by mitigating dedifferentiation and mesenchymal transition (Figure 5K–N; Figure S7E‐H, Supporting Information). In addition, podocyte apoptosis was markedly reduced in *Cre* ^+^/*Setdb2* ^AAV^ mice (Figure 5K,O).

Moreover, SETDB2 overexpression attenuated STZ/HFD‐induced SMAD3 upregulation and suppressed the acquisition of mesenchymal features in glomeruli (Figure 5P,Q). Furthermore, mitochondrial damage, evidenced by structural abnormalities, was ameliorated upon SETDB2 restoration in vivo (Figure 5R,S). Together, these results demonstrate that podocyte‐specific SETDB2 overexpression protects against podocytes dysfunction during DKD progression.

### 2.6 TCF21 Promotes SETDB2 Transcription by Directly Binding to Its Promoter

**Figure 6:**
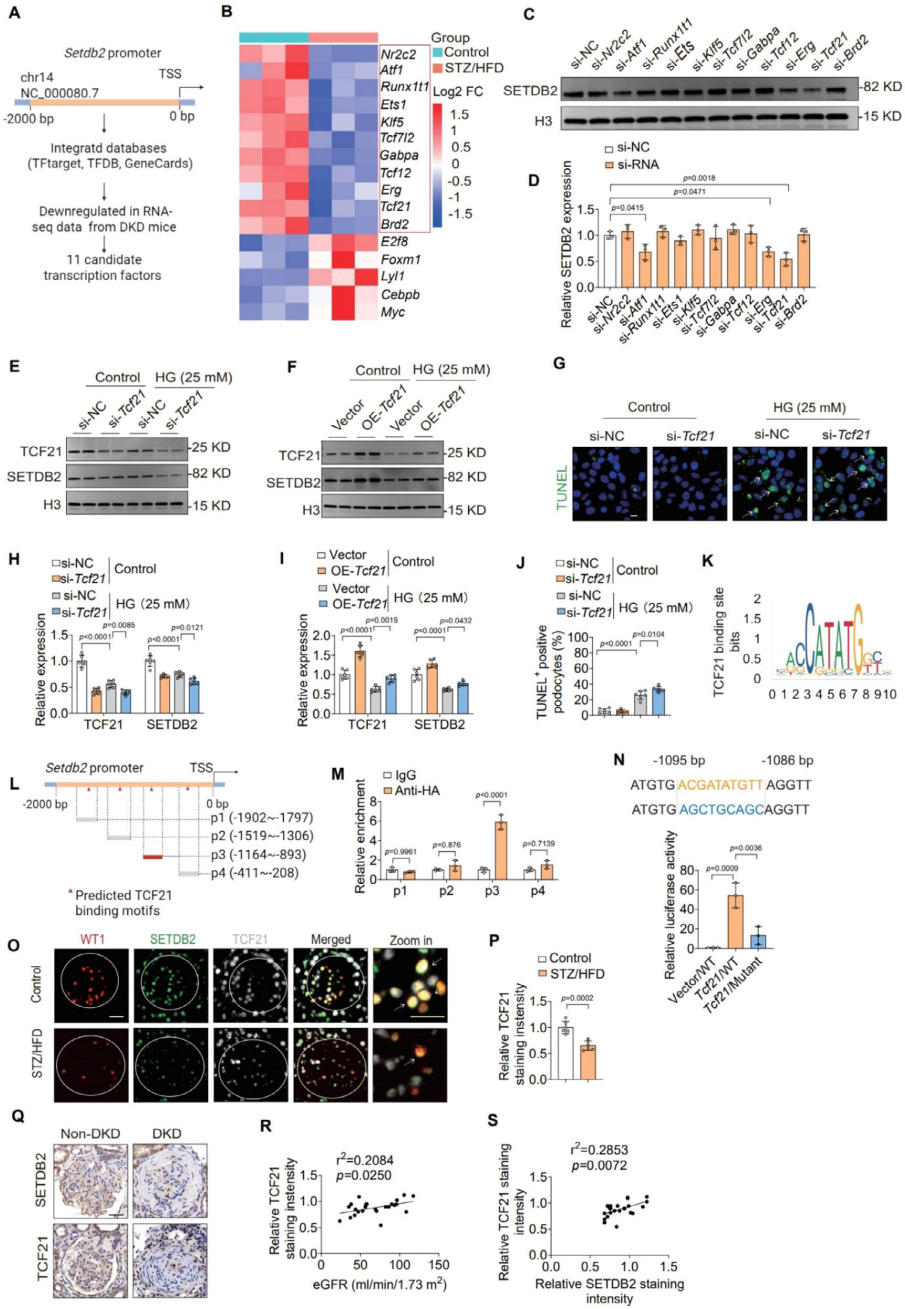
TCF21 promotes SETDB2 transcription by directly binding to its promoter. A) Schematic diagram of prediction of SETDB2‐promoter‐binding transcription factors (TFs) by integrating multiple TFs prediction databases with RNA‐seq data from glomeruli of DKD mice. TSS, transcription start site. B) Transcriptome sequencing was performed in glomeruli from *Cre* ^−^/*Setdb2* ^fl/fl^ and *Cre* ^+^/*Setdb2* ^fl/fl^ mice with STZ/HFD treatment. C,D) Representative Western blot images of SETDB2 expression in MPC5 cells transfected with 11 candidate siRNA for 48 h (n = 3). E,F/H,I) Representative Western blot images of SETDB2 and TCF21 expression and quantitative analysis in MPC5 cells with TCF21 silence or TCF21 overexpression treatments (n = 6). G/J) Representative IF images of TUNEL staining and quantitative analysis in MPC5 cells with TCF21 silence treatments (n = 6). Scale bar, 10 µm. K,L) Sequence logo depicting the putative TCF21 binding site, predicted using JASPAR software. M) ChIP‐qPCR with anti‐HA antibody demonstrated significant TCF21 binding to the *Setdb2* promoter regions in MPC5 cells compared to IgG control (n = 3). N) Luciferase reporter assays revealed that *Tcf21* overexpression enhanced WT but not mutant Setdb2 promoter activity. Schematic shows TCF21 binding site (blue) and mutated sequence (red) (n = 3). O,P) Representative IF images of TCF21 (white), WT‐1 (red) and SETDB2 (green) quantitative analysis of TCF21 expression in control and STZ/HFD mice (n  = 6). Scale bar, 20 µm(white), 20 µm (yellow). Q) Representative IHC images of SETDB2 and TCF21 in human renal tissues from non‐DKD controls (n  = 6) and moderate DKD patients (n  = 18). Scale bar, 50 µm. R) Correlation analysis between TCF21 expression and eGFR in human samples from non‐DKD controls (n  = 6) and DKD patients with varying disease severity (mild to severe) (n = 18). S) Correlation analysis between glomerular SETDB2 and TCF21 expression in human samples from non‐DKD controls (n = 6) and DKD patients with varying disease severity (mild to severe) (n = 18). Data are presented as Mean±SD. One‐way ANOVA with Tukey's post‐test (D, N), Two‐tailed Student's *t*‐test analysis (P, M), Two‐way ANOVA with Tukey's post‐test (H, I, J), with *p* values indicated.

To explore the upstream regulatory mechanisms governing SETDB2 transcription in podocytes, we predicted potential *Setdb2*‐promoter‐binding transcription factors (TFs) by integrating data from multiple prediction databases (TFtarget, TFDB, and GeneCards) with RNA‐seq data from glomeruli of DKD mice (**Figure** **6**A). This approach identified 11 candidate TFs potentially regulating *Setdb2*, including NR2C2, KLF5, RUNX1T1, ETS1, ATF1, TCF7L2, GABPA, TCF12, ERG, TCF21 and BRD2 (Figure 6B).

To validate these candidates, we performed siRNA‐mediated knockdown in MPC5 podocytes and assessed SETDB2 expression. Among the tested TFs, knockdown of *Atf1*, *Erg* and *Tcf21* significantly reduced SETDB2 protein levels, with knockdown *Tcf21* exerting the most pronounced effect (Figure 6C,D). Furthermore, *Tcf21* mRNA was significantly downregulated in HG‐induced MPC5 podocyte injury, whereas *Atf1* and *Erg* remained unchanged (Figure S8A, Supporting Information), suggesting that SETDB2 may be regulated by TCF21 in podocytes. Functionally, TCF21 knockdown exacerbated HG‐induced SETDB2 suppression, *Smad3* transcription and podocyte apoptosis (Figure 6E,H,G,J; Figure S8B, Supporting Information). In contrast, TCF21 overexpression restored SETDB2 levels and rescued podocyte dysfunction (Figure 6F,I; Figure S8C–E, Supporting Information). TGF‐β is a well‐established pathogenic driver of kidney disease development.^[^ ^24^ ^]^ Similarly, under TGF‐β stimulation, overexpression of TCF21 increased SETDB2 expression and consequently suppressed both the transcription and activation of SMAD3 (Figure S8F,G, Supporting Information). These findings further validate the pivotal role of the TCF21‐SETDB2‐SMAD3 axis in the pathogenesis of DKD.

To delineate the molecular mechanism of TCF21‐mediated SETDB2 regulation, we analyzed the *Setdb2* promoter using the JASPAR database, which predicted four putative TCF21 binding sites (−1902 to −1797 bp, −1519 to −1306 bp, −1164 to −893 bp, and −411 to −208 bp) (Figure 6K,L). ChIP‐qPCR analysis confirmed direct binding of TCF21 at −1164 to −893 bp, with significant enrichment compared to control IgG (Figure 6M). Functional luciferase reporter assays further revealed that TCF21 overexpression significantly enhanced *Setdb2* transcriptional activity, additionally, mutation of the putative binding site (−1095 to −1086 bp) markedly attenuated TCF21‐mediated transcription of *Setdb2*, substantially attenuated this activation, identifying this site as the critical regulatory element (Figure 6N).

Immunostaining in DKD mice showed decreased TCF21 expression and co‐localization of TCF21, SETDB2, and WT1 within glomeruli, supporting a podocyte‐specific regulatory axis (Figure 6O,P; Figure S8H–K, Supporting Information). Importantly, TCF21 expression was significantly reduced in glomeruli from DKD patients and strongly correlated with eGFR and SETDB2 levels (Figure 6Q–S). Together, these findings demonstrate that TCF21 directly binds to the *Setdb2* promoter region and transcriptionally activates its expression in podocytes. Loss of TCF21 contributes to SETDB2 downregulation and may represent an upstream event in DKD‐associated podocyte dysfunction.

## 3 Discussion

**Figure 7:**
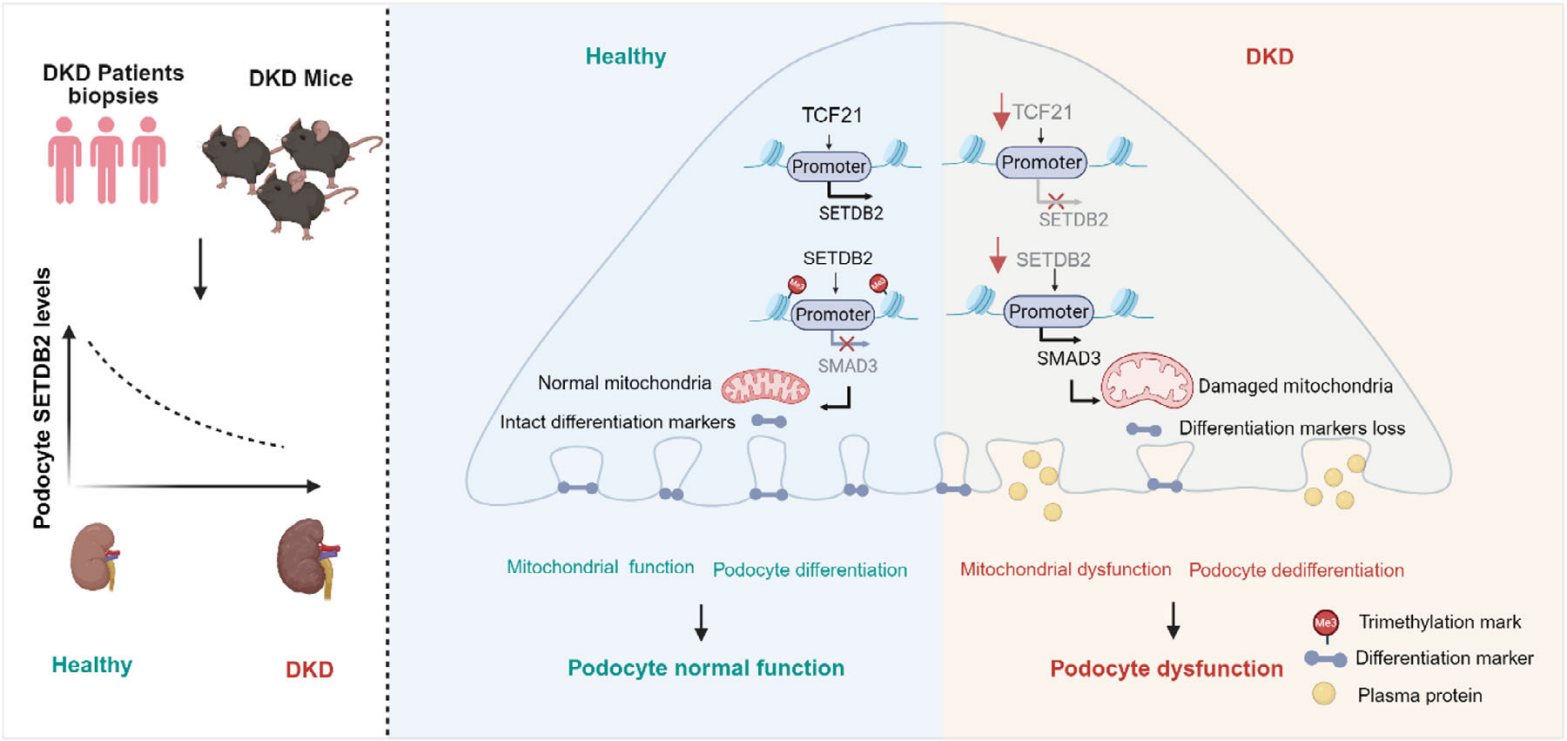
Schematic depicting SETDB2 protects against podocyte dysfunction in diabetic kidney disease. Created with BioRender.com (License ID: AW290EAWX4). SETDB2 epigenetically represses *Smad3* transcription by increasing H3K9me3 enrichment at its promoter, thereby mitigating podocyte dysfunction in DKD. Importantly, the transcription factor TCF21 binds directly to the *Setdb2* promoter and enhances its expression in podocytes.

Despite advances in DKD management, the disease continues to progress in a significant proportion of patients, emphasizing the critical necessity for elucidating its underlying mechanisms. Epigenetic regulation, which governs gene expression without alterations to DNA sequence, has emerged as a pivotal contributor to DKD pathogenesis.^[^ ^8^ ^]^ It mediates gene‐environment interactions that not only initiate and sustain renal injury, but also perpetuate disease‐related gene expression patterns even after normalization of glycemic control, a phenomenon referred to as metabolic memory.^[^ ^12^ ^]^ Among epigenetic regulators, chromatin‐modifying enzymes, chromatin‐modifying enzymes (“writers” and “erasers”) are particularly attractive therapeutic targets due to their dynamic and reversible nature.^[^ ^25^ ^]^ Notably, the therapeutic potential of targeting epigenetic enzymes has been successfully demonstrated in oncology, including small‐molecule inhibitors of DNMTs, HDACs, isocitrate dehydrogenase (IDH), and enhancer of zeste homolog 2 (EZH2) have been developed, and several have received regulatory approval.^[^ ^26^ ^]^ These precedents provide a strong rationale for repurposing or adapting epigenetic‐targeted approaches to non‐malignant chronic diseases, such as DKD. In this study, we identified the histone methyltransferase SETDB2, a “writer” of the repressive H3K9me3 mark, as a critical suppressor of podocyte dysfunction. Loss of SETDB2 resulted in elevated SMAD3 expression and activation, thereby aggravating glomerular damage and proteinuria in diabetic mice. These findings highlight SETDB2 as a previously unrecognized epigenetic regulator and a promising candidate for therapeutic intervention in DKD (**Figure** **7**).

Podocytes, as part of the glomerular filtration barrier, are indispensable for sustaining its structural integrity and selective permeability.^[^ ^6^ ^]^ Under diabetic conditions, podocytes are highly susceptible to injury due to their complex morphology and limited regenerative capacity.^[^ ^27^ ^]^ Sustained hyperglycemia and associated stressors trigger a cascade of pathological events, such as cytoskeletal disruption, loss of podocyte‐specific markers, dedifferentiation, EMT, and apoptosis, that collectively result in podocyte injury and dysfunction. These changes ultimately compromise the filtration barrier and contribute to proteinuria in DKD. Consequently, clarifying the mechanism underlying podocyte injury and dysfunction is critical for identifying effective therapeutic intervention. Increasing evidences have highlighted the importance of epigenetic modification, particularly histone modifications, in podocyte pathology. For instance, loss of the histone deacetylase SIRT6 leads to increased H3K9 acetylation and upregulation of *Notch1* and *Notch4*, exacerbating podocyte injury.^[^ ^28^ ^]^ Similarly, inhibition of the histone demethylases JMJD3 and UTX restores repressive H3K27me3 marks, thereby attenuating podocyte dedifferentiation.^[^ ^29^ ^]^ However, the function of HMTs in podocyte dysfunction is still unexplored. To address this gap, we conducted transcriptomic profiling of glomeruli from DKD mice and observed significant dysregulation of multiple HMTs. Among them, SETDB2 was the most prominently downregulated and showed an inverse correlation with disease severity in human renal biopsies. However, its functional role in DKD has not yet been investigated.

SETDB2 is a SET domain‐containing HMT that catalyzes the H3K9me3 mark, a key repressive epigenetic mark related to transcriptional silencing.^[^ ^14^ ^]^ It primarily targets transposable elements, satellite repeats, and gene promoters to maintain heterochromatin integrity. Although SETDB2 shares functional similarities with its homolog SETDB1, the two enzymes exhibit distinct biochemical and structural features. Their SET catalytic domains share only ∼36% sequence identity and differ markedly in their bifurcated architecture and inserted sequence motifs—differences that likely underlie their non‐redundant epigenetic roles.^[^ ^17^ ^]^ Unlike its homolog SETDB1, SETDB2 appears to exhibit substrate preference by converting H3K9me1 or H3K9me2 into H3K9me3. Although relatively understudied, SETDB2 has recently been implicated in immune regulation, metabolic diseases, and cancer.^[^ ^15^, ^17^, ^30^ ^]^ During acute influenza virus infection, SETDB2 is selectively induced in macrophages by type I interferon signaling via the STAT1–IRF7 axis.^[^ ^31^ ^]^ Once upregulated, SETDB2 deposits H3K9me3 at promoters of antiviral and proinflammatory genes, including *Mx1*, *Isg15*, and *Ccl2*, thereby suppressing their expression. This epigenetic repression serves to restrain innate immune activation and maintains immune homeostasis during infection. A similar interferon–SETDB2 regulatory axis operates in tissue repair. During wound healing, SETDB2 is induced by IFN‐β in macrophages and facilitates the shift from inflammatory state to reparative phenotype. Mechanistically, SETDB2 modifies the NF‐κB binding sites in the promoter regions of pro‐inflammatory genes via depositing H3K9me3 marks, leading to transcriptional suppression.^[^ ^16^ ^]^ In diabetes, impaired IFN‐β–SETDB2 signaling leads to unresolved inflammation and defective repair. Beyond its canonical role in histone methylation, SETDB2 has also been demonstrated to modulate gene expression through non‐histone mechanisms. During HFD–induced metabolic stress, SETDB2 expression is upregulated in hepatocytes, where it facilitates enhancer–promoter interactions at glucocorticoid receptor–responsive genes, such as *Insig2*, independent of H3K9me3 deposition.^[^ ^17^ ^]^ This scaffolding function contributes to the repression of lipogenic programs and protects against hepatic steatosis and insulin resistance. Although SETDB2 has been suggested to be involved in immune and metabolic regulation, its function in the kidney‐particularly in podocyte biology and DKD pathogenesis‐remains largely unexplored. In our study, we observed a marked reduction of SETDB2 expression in injured podocytes. Podocyte‐specific SETDB2 deficiency aggravated podocyte dysfunction and accelerated DKD progression, whereas targeted overexpression of SETDB2 using *Cre/loxP*‐mediated DNA inversion significantly alleviated podocyte dysfunction and improved glomerular damage in diabetic mice. These findings establish SETDB2 as a vital epigenetic mediator of structural and functional integrity of podocytes and a potential candidate for therapeutic intervention of DKD.

To investigate the mechanisms by which SETDB2 deficiency contributes to podocyte dysfunction, we performed RNA sequencing on glomeruli from podocyte‐specific *Setdb2* knockout diabetic mice. Transcriptomic profiling revealed substantial changes in pathways related to podocyte differentiation, EMT, inflammation, and apoptosis, key processes that underlie podocyte dysfunction and DKD progression. Functional validation confirmed that SETDB2 deficiency aggravated dedifferentiation and apoptosis in podocytes, which was accompanied by marked mitochondrial abnormalities, including swelling, fragmentation, and disrupted cristae structure as revealed by transmission electron microscopy, indicative of impaired mitochondrial integrity and energy metabolism. These findings further support the role of epigenetic dysregulation in podocyte dysfunction.

As a histone methyltransferase, SETDB2 is presumed to exert its regulatory effects primarily through chromatin remodeling. However, its downstream targets in DKD remained undefined. To address this, we employed CUT&Tag, a high‐sensitivity method for mapping histone modifications and transcription factor binding. Integration of CUT&Tag data with transcriptomic results identified *Smad3*, a key regulatory molecule involved in renal fibrosis and inflammation,^[^ ^32^ ^]^ as a direct epigenetic target of SETDB2, repressed via H3K9me3 deposition at its promoter region. Except for *Smad3*, SETDB2 and H3K9me3 signals were also altered at the promoters of several fibrosis‐and inflammation‐related genes including *Snai1*, *Col18a1*, *Cd86*, *Ccl2*; however, RNA‐seq analysis showed no corresponding changes in the transcriptional levels of these genes. Therefore, we infer that SETDB2 does not directly modulate H3K9me3 deposition at these genes but may instead influence their transcription through indirect regulatory mechanisms. Furthermore, following *Setdb2* deletion, several genomic regions exhibited SETDB2 enrichment patterns that were distinct from H3K9me3 distribution, such as the *Il11* and *Pdgfa* loci. These observations indicate that SETDB2 may also exert additional regulatory functions independent of its canonical H3K9me3‐associated activity under pathological conditions.

Among receptor‐regulated SMADs, SMAD2 and SMAD3 are the principal downstream effectors activated by multiple upstream stimuli. Upon phosphorylation, SMAD2/3 associates with the common mediator SMAD4 to form a heteromeric complex, and this complex migrates into the nucleus to activate the transcription of genes involved in fibrosis.^[^ ^33^ ^]^ In DKD, SMAD3 has been identified as a key pathogenic effector that integrates multiple profibrotic signals, including advanced glycation end products (AGEs), angiotensin II (Ang II), TGF‐β, and C‐reactive protein (CRP). Unlike SMAD2, SMAD3 can directly bind DNA via its MH1 domain, enabling it to regulate a broader set of target genes.^[^ ^22^ ^]^ Genetic deletion of *Smad3* significantly reduces proteinuria in HFD‐induced diabetic models, highlighting its pathological relevance.^[^ ^34^ ^]^ Mechanistically, SMAD3 governs transcriptional programs associated with extracellular matrix deposition, EMT, and mitochondrial dysfunction.^[^ ^24^, ^35^, ^36^ ^]^ During EMT, SMAD3 promotes the expression of collagens (such as Col1a1) and other extracellular matrix (ECM) or mesenchymal genes, while facilitating the repression of epithelial markers (such as E‐cadherin) through the induction of EMT‐related transcription factors (e.g., Snai1), thereby driving the EMT process.^[^ ^35^, ^37^ ^]^ Concurrently, SMAD3 impairs mitochondrial homeostasis by suppressing PGC‐1α‐dependent biogenesis,^[^ ^36^ ^]^ inducing NOX4‐derived ROS,^[^ ^38^ ^]^ and promoting mitochondrial fission,^[^ ^39^ ^]^ leading to mitochondrial swelling, cristae disruption, and bioenergetic dysfunction.

In our study, we identified *Smad3* as a direct transcriptional target of the histone methyltransferase SETDB2. We demonstrated that SETDB2 represses *Smad3* expression through H3K9me3‐mediated chromatin silencing, thereby mitigating podocyte dedifferentiation and dysfunction. Importantly, *Smad3* silencing reversed the detrimental effects of SETDB2 deficiency, establishing the SETDB2–SMAD3 regulatory axis as a critical epigenetic mechanism in podocyte protection. Numerous studies have shown that SMAD3 activity is tightly regulated through multiple mechanisms. For instance, the E3 ubiquitin ligase NEDD4L could catalyze the ubiquitination and subsequent proteasomal degradation of phosphorylated SMAD3, thereby limiting its nuclear translocation and downstream signaling Similarly, the dual‐specificity phosphatase DUSP1 dephosphorylates SMAD3 and suppresses its activity in an HDAC1‐acetylation–dependent manner. Given that TGFβ‐SMAD3 signaling is and initiated by TGF‐β ligand binding to TβRII/TβRI followed by TβRI‐mediated phosphorylation of SMAD3.^[^ ^40^ ^]^ However, loss of SETDB2 did not significantly affect the expression of major regulators involved in SMAD3 post‐translational modifications. Interestingly, SETDB2 deficiency notably increased TGF‐β1 secretion from podocytes, the principal upstream activator of SMAD3. These results suggest that SETDB2 loss primarily enhances SMAD3 transcription by reducing H3K9me3 deposition at its promoter, whereas SMAD3 hyperactivation may also result, at least in part, from elevated TGF‐β1 levels. Of course, we acknowledge that multiple factors are involved in regulating SMAD3 post‐translational modifications, and our current analyses cannot comprehensively capture all relevant pathways. Therefore, we do not exclude the possibility that other diabetes‐related molecules may also modulate SMAD3 activity. In addition, the TGF‐β1 secreted from podocytes may not only act in an autocrine manner to reinforce their own profibrotic and dedifferentiation responses, but also exert paracrine effects on neighboring glomerular cells, including endothelial and mesangial cells, which trigger a cascade of signaling events to exacerbate glomerular injury, disrupt filtration barrier integrity, and accelerate DKD progression. These findings not only broaden our insights into SMAD3 regulation in DKD but also suggest novel therapeutic opportunities targeting this axis.

According to previous studies, SETDB2 expression can be induced by interferon signaling in diabetic wound macrophages.^[^ ^15^, ^41^ ^]^ In contrast, our findings show that high glucose exposure results in a substantial decrease in SETDB2 expression in podocytes, suggesting a cell type–specific regulatory pattern. However, the upstream molecular mechanisms governing this downregulation remain elusive. To address this, we analyzed the *Setdb2* promoter sequence and identified TCF21, a basic helix‐loop‐helix (bHLH) transcription factor, as a candidate regulator. This prediction was validated by a pronounced positive correlation between TCF21 and SETDB2 expression in renal biopsies from DKD patients. TCF21 is known to regulate podocyte differentiation and glomerular development by modulating cytoskeletal components and maintaining slit diaphragm architecture, often in cooperation with WT1.^[^ ^42^ ^]^ Functionally, TCF21 is required during podocyte development, maturation, and injury responses, partly through its influence on Wnt/β‐catenin and Notch signaling pathways.^[^ ^43^, ^44^ ^]^ Notably, decreased TCF21 expression has been observed in diabetic kidneys, and podocyte‐specific *Tcf21* knockout mice manifest signs of early‐onset proteinuria and glomerular lesions, underscoring its protective role in glomerular homeostasis.^[^ ^45^ ^]^ In this study, we demonstrated that TCF21 directly regulates SETDB2 expression, thereby modulating podocyte dedifferentiation and markedly attenuated SMAD3 activation even in the presence of TGF‐β. These findings reveal transcription factor TCF21 as a novel upstream regulator of SETDB2 and broaden its functional role in maintaining podocyte identity in the setting of DKD.

There were several limitations to this study. First, C57BL/6 mice are relatively resistant to developing overt DKD,^[^ ^46^, ^47^ ^]^ and pronounced renal lesions usually require additional renal injury procedures (such as uninephrectomy).^[^ ^28^ ^]^ Moreover, a single animal model cannot fully recapitulate the complex pathophysiology of human DKD. Thus, the role of SETDB2 warrants further validation across multiple animal models (e.g., mice, rats, and non‐human primates). Second, although our integrative transcriptomic and epigenomic analyses identified SMAD3 as a direct target of SETDB2 through H3K9me3 deposition, we did not investigate the potential non‐histone substrates of SETDB2 in podocytes. Given emerging evidence that SETDB2 may regulate cellular function through alternative methylation‐independent scaffolding or non‐histone methylation mechanisms, its broader epigenetic repertoire in podocyte biology warrants further exploration. Third, AAV‐mediated re‐expression of SETDB2 in podocytes effectively ameliorated diabetic kidney injury, highlighting its therapeutic potential. However, the long‐term durability and safety of AAV‐based approaches remain to be further evaluated in preclinical and clinical settings. In addition, pharmacological modulation of SETDB2 activity through small molecules or epigenetic regulators may represent a more clinically feasible strategy, warranting future investigation. Translating these findings into clinical applications will require rigorous evaluation in human studies.

In conclusion, our findings establish SETDB2 as a previously unrecognized epigenetic safeguard of podocyte identity, acting through H3K9me3‐mediated repression of SMAD3 to counteract diabetic injury. This study not only expands the current understanding of chromatin regulation in glomerular pathology but also positions SETDB2 as a compelling candidate for therapeutic modulation in diabetic kidney disease.

## 4 Experimental Section

Detailed materials and methods are described in the Supplementary Information and Supplementary Methods.

### Human Renal Samples

The specimens were provided by the Nephrology Department at China‐Japan Friendship Hospital. Human renal biopsy samples were obtained during standard diagnostic procedures, with clinical characteristics documented in Table S1 (Supporting Information). The study included 15 control samples derived from renal tissues adjacent to tumors in nephrectomized patients, all of whom had no history of diabetes or renal disorders. Additionally, 45 renal samples were collected from DKD patients, classified according to the Renal Pathology Society's pathological criteria. This study received approval from the Ethics Committee of the China‐Japan Friendship Hospital (Approval No. 2024‐KY‐129‐1) and complied with the ethical guidelines of the Declaration of Helsinki. All participants provided written informed consent before sample collection.

### Animal Studies

All animal experiments were approved by the Animal Ethics Committee of China‐Japan Friendship Hospital (Approval No. ZRDWLL240126). All procedures were carried out in compliance with the National Institutes of Health guidelines for laboratory animal welfare.

### Generation of Podocyte‐Specific Setdb2 Knockout Mice

Podocyte‐specific *Setdb2* knockout (*Nphs2*‐*Cre* ^+^ / *Setdb2* ^flox/flox^) mice were produced by hybridizing *Nphs2*‐*Cre* mice and *Setdb2^flox/flox^* mice. (Cyagen Biosciences, Suzhou, China). Control groups consisted of *Setdb2* ^flox/flox^ mice lacking Cre recombinase expression (*Setdb2* ^flox/flox^ mice; *Nphs2*‐*Cre* ^−^/*Setdb2* ^flox/flox^ mice). Genomic DNA extracted from tail biopsies at postnatal week 2 was analyzed by PCR for genotype verification.

### Isolation of Glomeruli

Glomeruli were isolated following a refined protocol adapted from multiple published references.^[^ ^48^, ^49^ ^]^ Briefly, after anesthesia, mice underwent vascular ligation to establish a closed renal circuit. Followed by perfusion with Dynabeads (8 × 10⁷ beads per mouse; Invitrogen) in warm HBSS (37 °C). Kidneys were excised, minced, and digested (collagenase A, 37 °C, 1 h). The digested tissue was filtered by cell strainer (70 µm), washed with HBSS, and centrifuged (200 g, 5 min, 4 °C). Dynabead‐bound glomeruli were isolated magnetically after three HBSS washes. The purified glomeruli were for RNA‐seq analyses and stored at −80 °C for further verification analyses.

### CUT and Tag Assay

CUT&Tag assay was carried out by using Hyperactive Universal CUT&Tag Assay Kit for Illumina (Cat. No. TD904‐01, Vazyme Biotech, China). Briefly, cells were bound to ConA‐coated magnetic beads. Permeabilization was performed with digitonin. Sequential incubations were carried out with primary antibody (SETDB2 and H3K9me3), secondary antibody and hyperactive pA‐Tn5 Transposase. Tn5‐mediated DNA fragmentation and adapter ligation (P5/P7) were performed in situ. Libraries were PCR‐amplified, purified, and quality‐checked (Agilent 2100 Bioanalyzer). Illumina NovaSeq6000 was employed for sequencing. The raw data were initially subjected to quality trimming using fatsq software. Subsequently, Bowtie2 was utilized to map the clean reads to the GRCm39 reference genome. IGV was used to visualize peak distribution in genomic regions of interest. ChIPseeker was then employed to annotate peaks and identify corresponding genes. MAnorm was applied to analyze differential peaks between conditions.

### Screening for Transcription Factors Bind with the Setdb2 Promoter

Based on either strand of *Setdb2* promoter sequences, multiple prediction databases (TFtarget, TFDB, and GeneCards) was integrated analysis for screening the transcription factors. The 16 candidate TFs potentially regulating *Setdb2* and 11 transcription factors were consistent with the reduction of SETDB2 in DKD sample.

### Statistical Analysis

Data are presented as Mean ± standard deviation (SD) and analyzed by GraphPad Prism software (v9.5, San Diego, CA). The correlation analysis was conducted using Pearson's correlation coefficients. Inter‐group differences were analyzed by: (1) two‐tailed Student's t‐tests were for pairwise comparisons, (2) one‐way ANOVA with Tukey's multiple comparison analysis between multiple groups with single variable, (3) two‐way ANOVA with Tukey's multiple comparison analysis between multiple groups with multivariable. The *p* values have been indicated in the figure and statistical significance was considered when *p* < 0.05.

## Conflict of Interest

The authors declare no conflict of interest.

## Author Contributions

L.L., S.J., Q.J., and P.Q. contributed equally to this work. L.L., S.J., and Q.J. designed and carried out experiments, analyzed data, and drafted the manuscript. L.L., S.J., and Q.J. collected and analyzed human samples. L.L. and P.Q. revised the paper according to reviewer comments. P.Q. and X.Y.L. carried out in vitro experiments. Y.G., C.S., and X.S.L. performed molecular biology experiment. X.L. performed the confocal microscopy. W.S., S.A., and J.G. performed histological analysis of renal tissues. W.L., L.P., and H.L. designed the studies, provided sources of the studies and participated in drafting the manuscript. The final version of the manuscript has been reviewed and consented to by all authors.

## Supporting information



Supporting Information

Supplemental Source Data 1

## Data Availability

The data that support the findings of this study are available in the supplementary material of this article.
