# Supplementary material for: SETDB2 Mitigates Podocyte Dysfunction in Diabetic Kidney Disease Through Epigenetic Silencing of SMAD3
Source: Adv Sci (Weinh). 2025 Nov 29;13(9):e16984. doi: 10.1002/advs.202516984 (PMC12904062; doi:10.1002/advs.202516984)
Supplement: Supplementary file 1 — Supporting Information [file ADVS-13-e16984-s002.docx]

**Supplementary Information**

**SETDB2 mitigates podocyte dysfunction in diabetic kidney disease through epigenetic silencing of SMAD3**

Lanfang Li^1, #^, Shimin Jiang^2, #^, Qi Jin^3, #^, Peng Qu^1#^, Yingjie Guo^1^, Xushan Lan^1^, Xinyu Li^1^, Cuiting Sun^4^, Sinan Ai^5^, Xin Li^1^, Weiliang Sun^1^, Jing Guo^1^, Wenge Li^2,^ *, Lihong Liu^1,6,7,^ *, Liang Peng^1,6,^ *

**Affiliations**

^1^ Beijing Key Laboratory for Immune-Mediated Inflammatory Diseases, Institute of Clinical Medical Sciences, China-Japan Friendship Hospital, Beijing 100029, China.

^2^ Department of Nephrology, China-Japan Friendship Hospital, Beijing 100029, China.

^3^ Guang'anmen Hospital, China Academy of Chinese Medical Sciences, Beijing 100053, China.

^4^ China-Japan Friendship Hospital, Capital Medical University, Beijing, 100000, China.

^5^ Diabetes Department of integrated Chinese and Western medicine, China-Japan Friendship Hospital, Beijing 100029, China.

^6^China-Japan Friendship Hospital (Institute of Clinical Medical Sciences), Chinese Academy of Medical Sciences & Peking Union Medical College, Beijing 100730, China.

^7^ Department of Pharmacy, China-Japan Friendship Hospital, Beijing 100029, China.

***** **Corresponding author**

liwenge@pumc.edu.cn (W.L.)

liulihong@zryhyy.com.cn (L.L.)

pengliang@zryhyy.com.cn (L.P.)

^#^ Contributed equally.


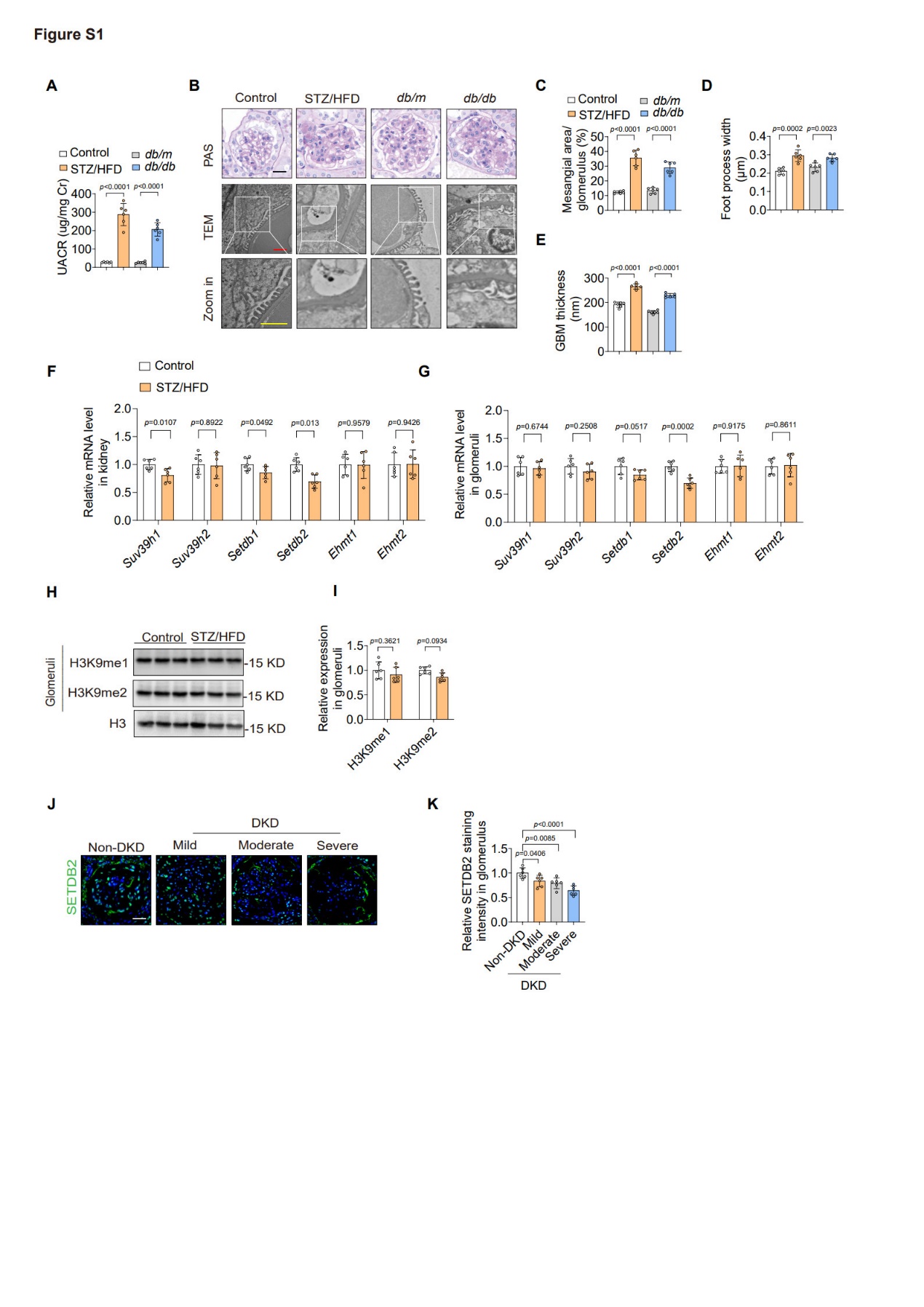


**Figure S1. SETDB2 and H3K9me3** **was significantly reduced in the kidney from DKD mice. Related to Figure 1.**

(A) UACR in different groups of mice (n = 6).

(B-E) Representative images of PAS and TEM staining and quantitative analysis in glomeruli from different groups of mice (n = 6). Scale bar, 20 μm (black), 1 μm (red), 1 μm (yellow).

(F-G) Relative mRNA level of KMT1-family members in the kidney and glomeruli from control and STZ/HFD mice (n = 6).

(H-I) Representative Western blot images of H3K9me1 and H3K9me2 expression and quantitative analysis in glomeruli from control and STZ/HFD mice (n = 6).

(J-K) Representative IF images of SETDB2 and quantitative analysis in human renal tissues from non-DKD controls and DKD patients with varying disease severity (mild to severe). Scale bar, 50 μm.

Data are presented as Mean±SD. Two-tailed Student’s unpaired t-test analysis (A, C-G, I), One-way ANOVA with Tukey’s post-test (K), with *p* values indicated.


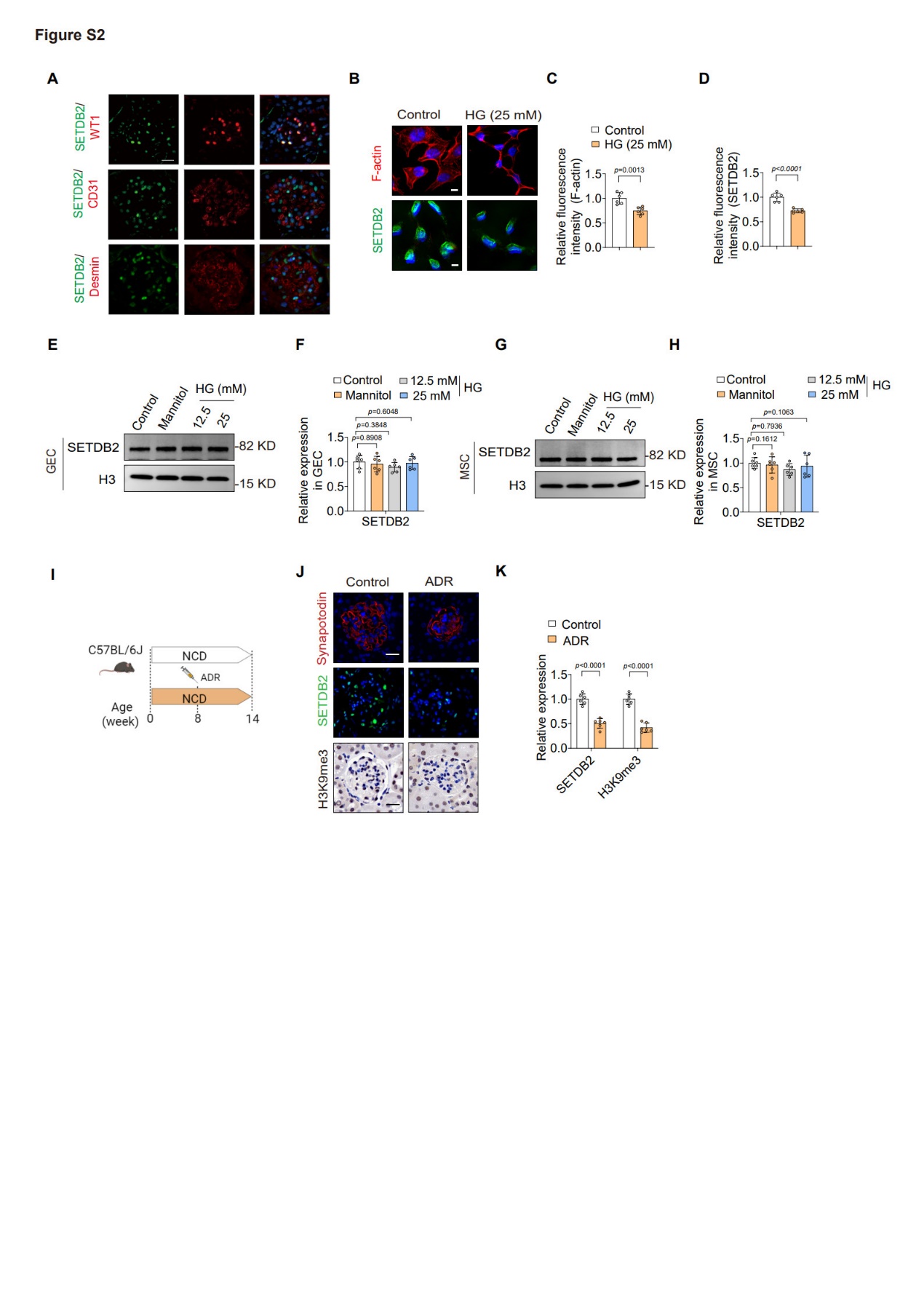


**Figure S2. Podocyte SETDB2 was significantly reduced under HG condition.** **Related to Figure 1.**

(A) Representative confocal microscopic images showing the SETDB2 (green) and WT-1 (podocyte marker, red), CD31 (endothelial cells marker, red), and desmin (mesangial cells marker, red) staining from control mice (n = 6). Scale bar, 20 μm.

(B-D) Representative IF images of SETDB2 (green) and F-actin (by phalloidine, red) staining and quantitative analysis in mouse podocyte cell line 5 (MPC5) cells in the presence or absence of HG (n = 6). Scale bar,10 μm.

(E-H) Representative Western blot images of SETDB2 expression and quantitative analysis in glomerular endothelial cells (GEC) and mesangial cells (MSC) in the presence or absence of HG (n = 6).

(I) Schematic representation of the experimental design of adriamycin (ADR)-induced nephropathy mouse models (Created in BioRender, https://www.biorender.com).

(J-K) Representative images of Synaptopodin (IF), SETDB2(IF), H3K9me3(IHC) and quantitative analysis in Adriamycin (ADR)-induced nephropathy mouse models. Scale bar,20 μm.

Data are presented as Mean±SD. Two-tailed Student’s unpaired t-test analysis (C, D, K), One-way ANOVA followed by Tukey’s post-test (F, H), with *p* values indicated.


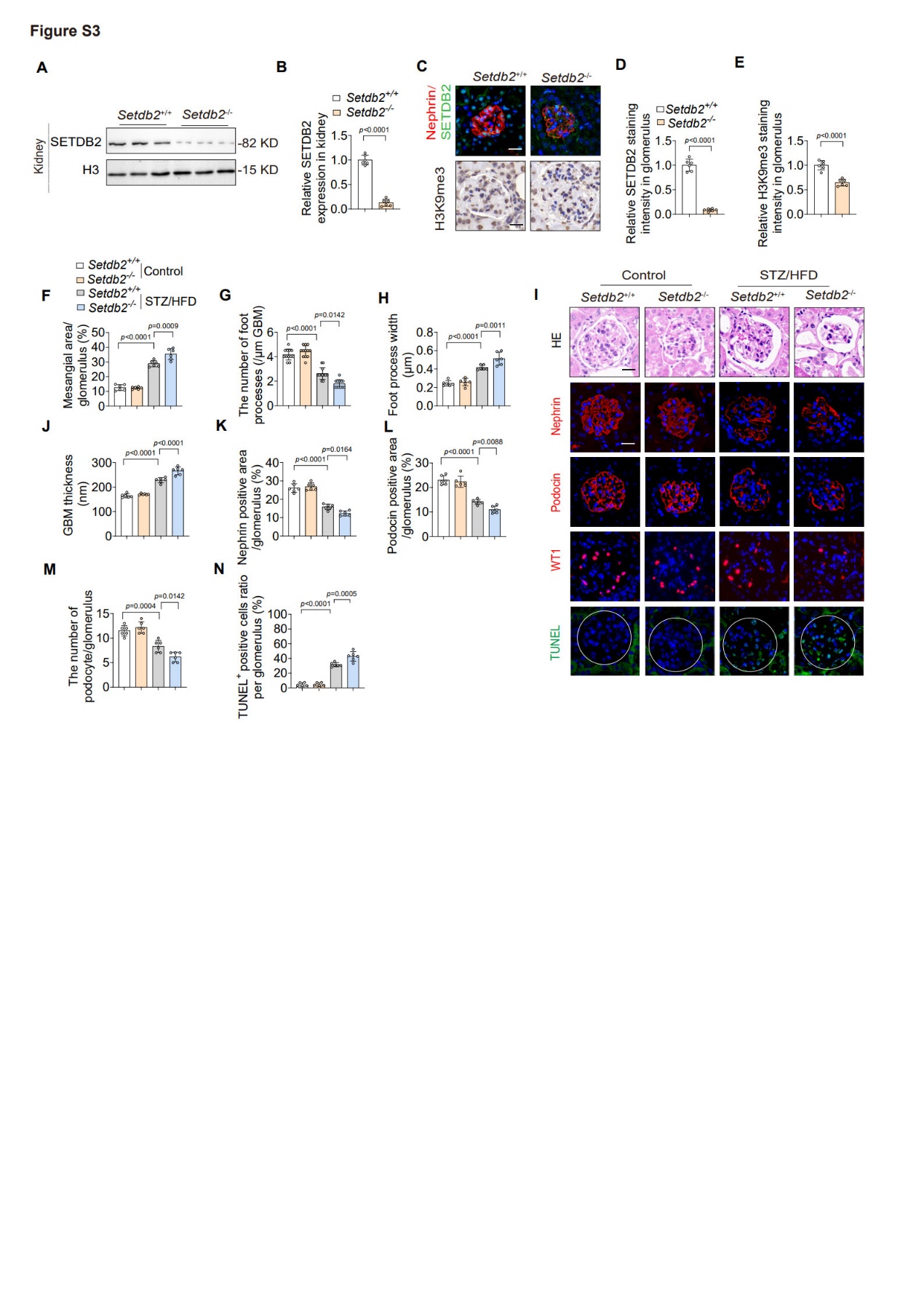


**Figure S3.** **Homozygotes *Setdb2* deficiency in mice deficiency aggravates DKD progression. Related to Figure 2.**

(A-B) Representative Western blot images of SETDB2 expression and quantitative analysis in kidney from *Setdb2*^+/+^ and *Setdb2*^-/-^ mice (n = 6).

(C-E) Representative images of Nephrin/SETDB2(IF), H3K9me3 (IHC) and quantitative analysis in *Setdb2*^+/+^ and *Setdb2*^-/-^ mice (n = 6). Scale bar, 20 μm.

(F-J) Quantitative analysis of TEM staining in *Setdb2^+/+^* and *Setdb2^-/-^* mice with control or STZ/HFD treatment (n = 6).

(K-I) Representative images of HE, Nephrin, Podocin, WT-1 and TUNEL(IF) staining and quantitative analysis in glomeruli from *Setdb2*^+/+^ and *Setdb2*^-/-^ DKD mice. Scale bar, 20 μm.

Data are presented as Mean±SD. Two-tailed Student’s unpaired t-test analysis (B, D, E), Two-way ANOVA followed by Tukey’s post-test (F-H, J-N), with *p* values indicated.


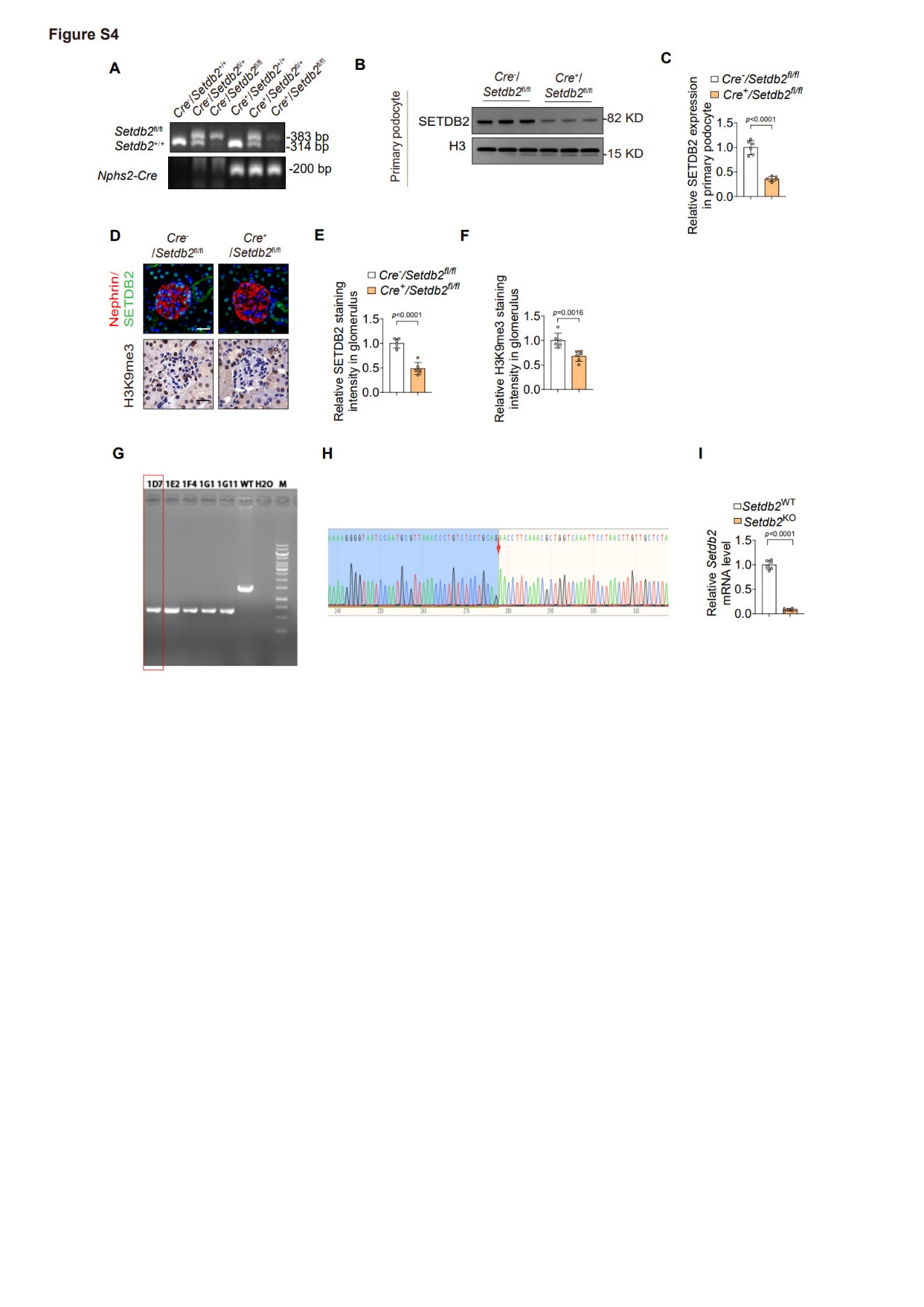


**Figure S4. Podocyte-specific SETDB2 deficiency aggravates DKD progression.** **Related to Figure 2.**

(A) Genotyping of different groups of mice was confirmed by tail preparation and PCR at 2 weeks of age.

(B-C) Representative Western blot images of SETDB2 expression and quantitative analysis in primary podocytes from *Cre*^-^/*Setdb2*^fl/fl^ and *Cre*^+^/*Setdb2*^fl/fl^ mice (n =6).

(D-F) Representative images of Nephrin/SETDB2(IF), and H3K9me3 (IHC) and quantitative analysis in *Cre*^-^/*Setdb2*^fl/fl^ and *Cre*^+^/*Setdb2*^fl/fl^ mice (n = 6). Scale bar, 20 μm.

(G-H) The PCR identification and sequencing results of knockout the mouse Setdb2 gene (*Setdb2*^KO^) in the MPC5 cells.

(I) Relative *Setdb2* mRNA level in *Setdb2*^WT^ and *Setdb2*^KO^ MPC5 cells (n = 6).

Data are presented as Mean±SD. Two-tailed Student’s unpaired t-test analysis (C, E, F, I), with *p* values indicated.


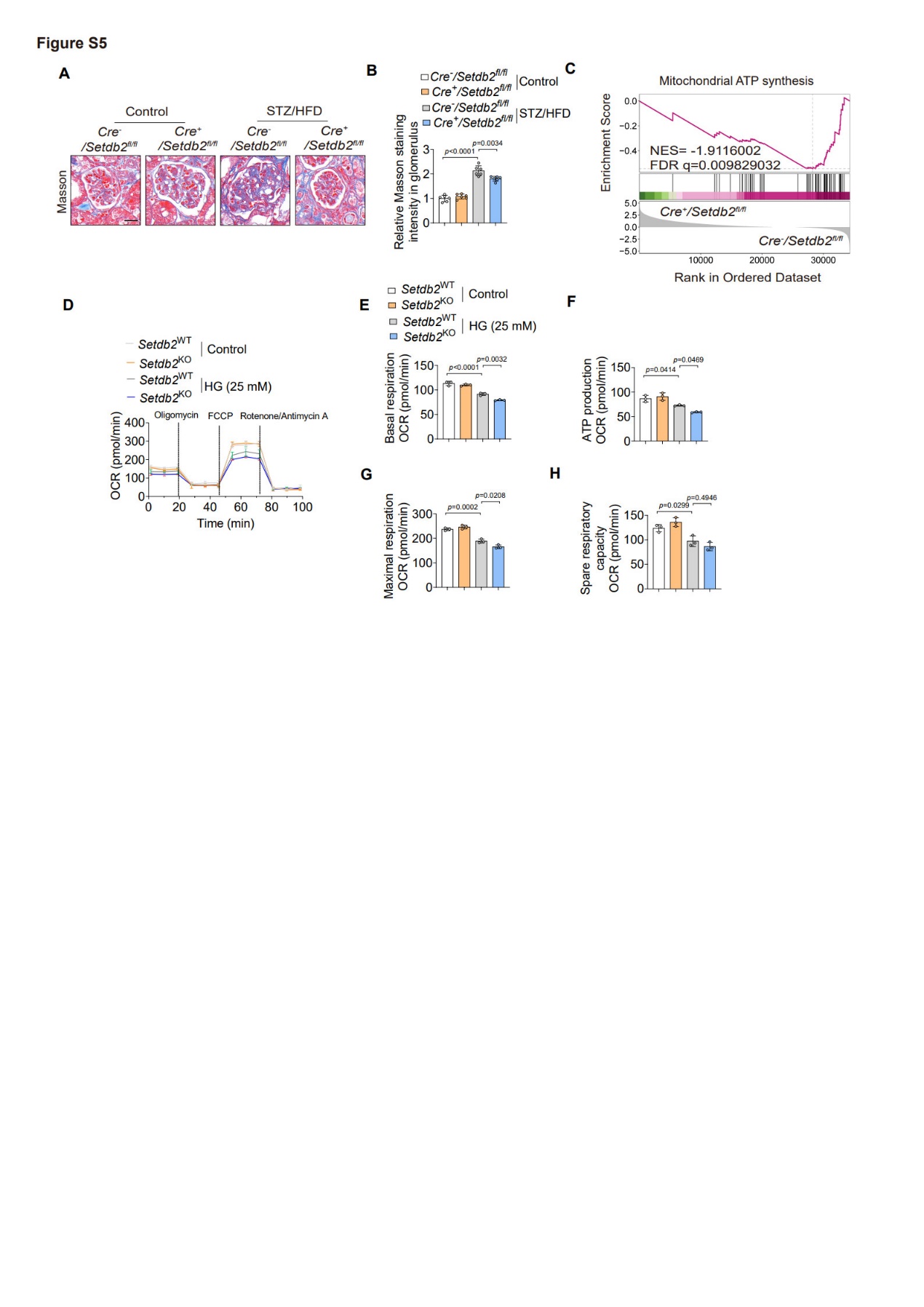


**Figure S5. Podocyte-specific SETDB2 deficiency promotes podocyte dysfunction in DKD mice.** **Related to Figure 3.**

(A-B) Representative Masson staining images of collagen deposition (Masson staining) and quantitative analysis in *Cre*^-^/*Setdb2*^fl/fl^ and *Cre*^+^/*Setdb2*^fl/fl^ mice with control and STZ/HFD treatment (n = 6). Scale bar: 20 μm.

(C) GSEA of mitochondrial ATP synthesis pathways were significantly enriched in *Cre*^-^/*Setdb2*^fl/fl^ mice with STZ/HFD treatment, compared to the *Cre*^+^/*Setdb2*^fl/fl^ mice with STZ/HFD treatment.

(D-H) Mitochondrial respiration profiles of *Setdb2*^KO^ and *Setdb2*^WT^ MPC5 cells in the presence or absence of HG as determined by the Seahorse analyzer and MitoStress assay (n = 3).

Data are presented as Mean±SD. Two-way ANOVA followed by Tukey’s post-test (B, E-H), with *p* values indicated.


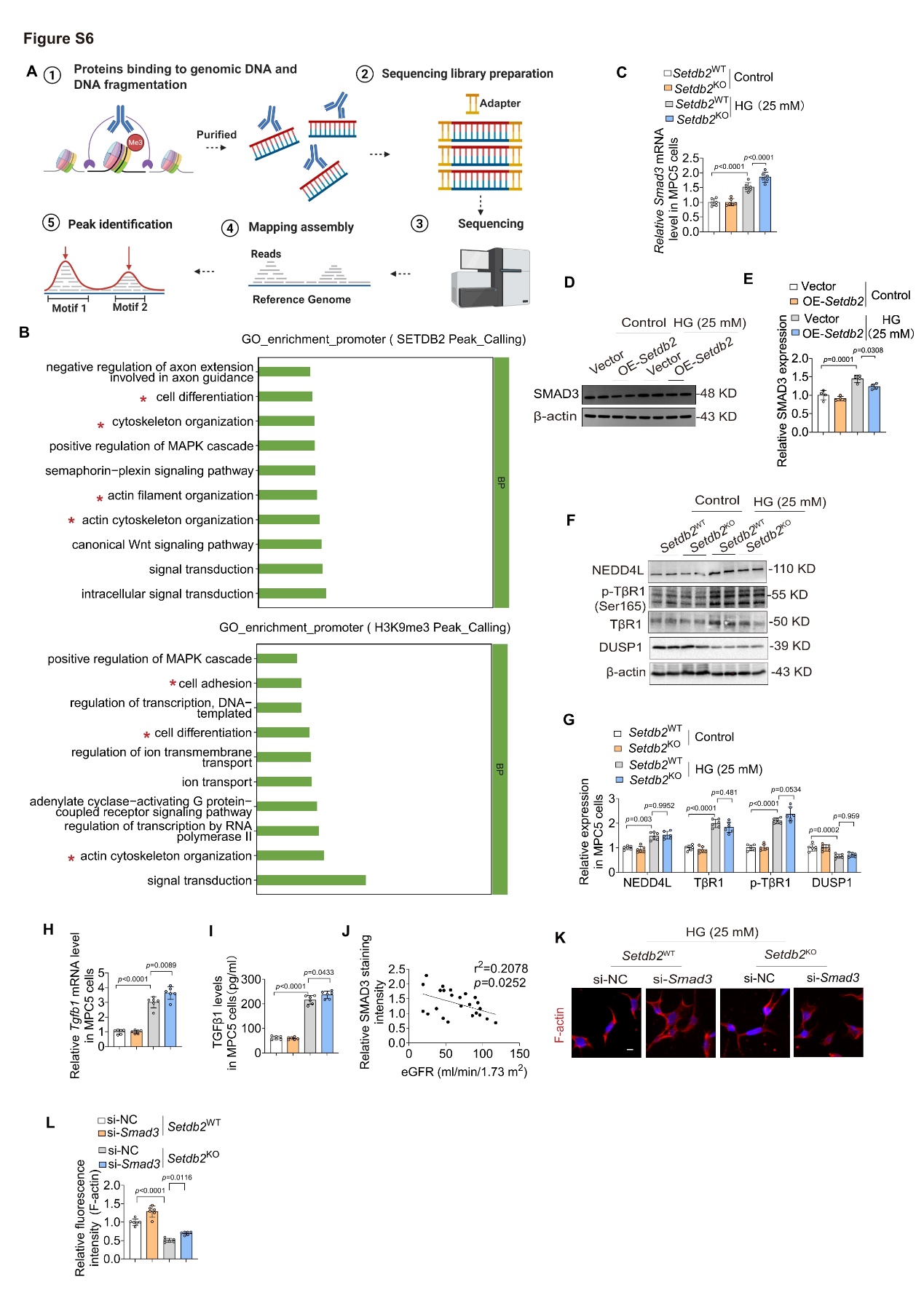
**Figure S6. SETDB2 deficiency promotes podocytes dysfunction via H3K9me3-dependent activation of SMAD3.** **Related to Figure 4.**

(A) The schematic diagram of CUT&Tag assay used by H3K9me3 and SETDB2 antibody in *Setdb2*^WT^ and *Setdb2*^KO^ MPC5 cells with HG treatment (Created in BioRender, https://www.biorender.com).

(B) GO analyses of the DEGs.

(C) Relative mRNA level of *Smad3* in *Setdb2*^WT^ and *Setdb2*^KO^ MPC5 cells with the presence or absence of HG (n=6).

(D-E) Representative Western blot images of SMAD3 expression and quantitative analysis in overexpression *Setdb2* in MPC5 cells with the presence or absence of HG (n=4).

(F-G) Representative Western blot images of TβR1, phospho-TβR1 (SerS165), NEDD4L and DUSP1 expression and quantitative analysis in *Setdb2*^WT^ and *Setdb2*^KO^ MPC5 cells with the presence or absence of HG (n=6).

(H-I) Relative mRNA level of *Tgfb1* and TGF-β1 concentration in *Setdb2*^WT^ and *Setdb2*^KO^ MPC5 cells with the presence or absence of HG (n=6).

(J) Correlation analysis between glomerular SMAD3 expression and eGFR in human subjects (n=24).

(K-L) Representative IF images of F-actin staining and quantitative analysis in *Setdb2*^KO^ and *Setdb2*^WT^ MPC5 cells with si-NC or si-*Smad3* intervention (n = 6). Scale bar, 10 μm.

Data are presented as Mean±SD. Two-way ANOVA followed by Tukey’s post-test (C, E, F, G, I, L), with *p* values indicated.


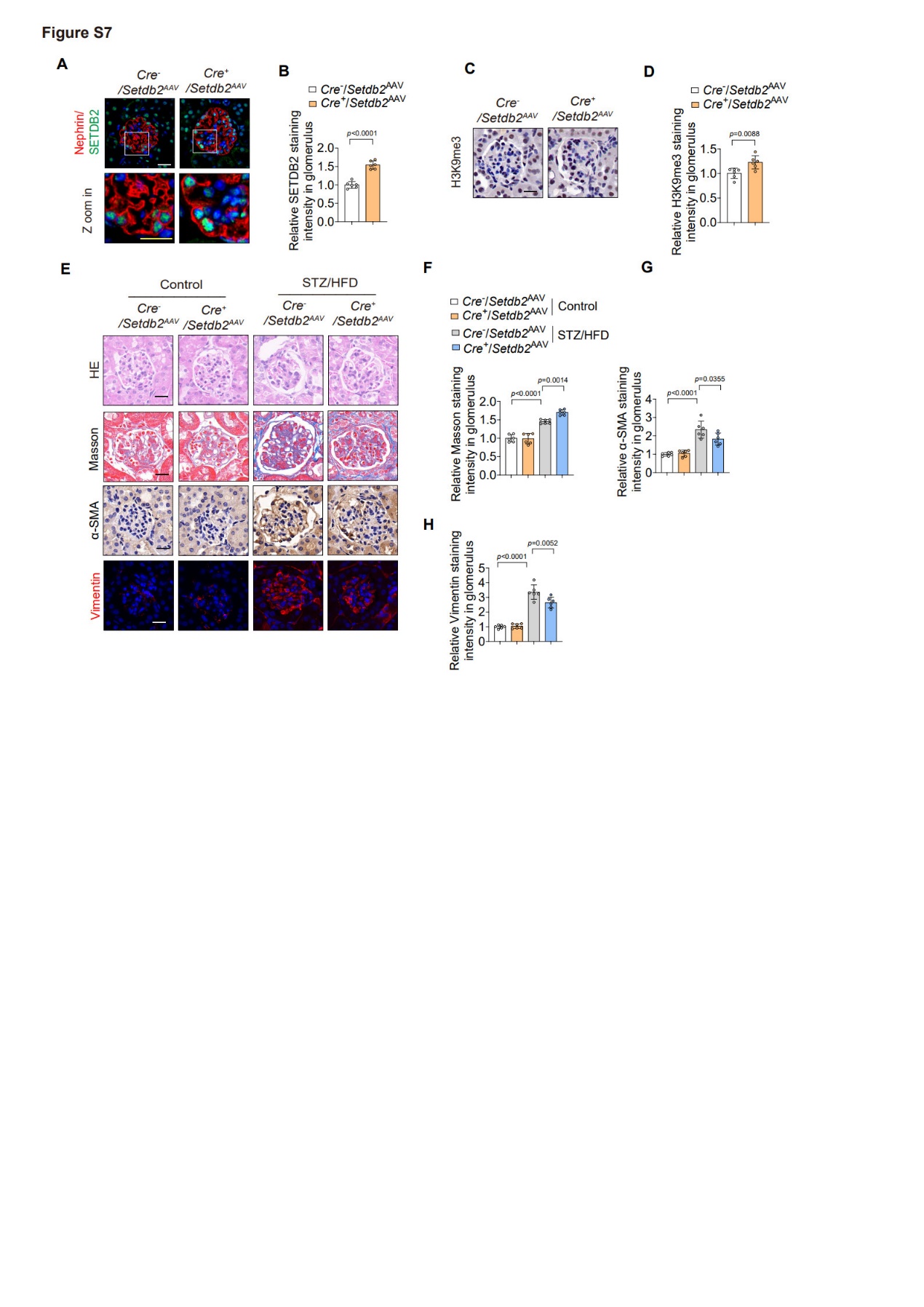


**Figure S7. Podocyte-specific SETDB2 overexpression alleviates podocyte dysfunction in DKD. Related to Figure 5.**

(A-B) Representative confocal microscopic images showing the expression of Nephrin (red) and SETDB2 (green) in glomeruli from *Cre*^-^/*Setdb2 ^AAV^* and *Cre*^+^/*Setdb2 ^AAV^* mice (n = 6). Scale bar, 20 μm(white), 20 μm (yellow).

(C-D) Representative IHC images of H3K9me3 and quantitative analysis in glomeruli from *Cre*^-^/*Setdb2 ^AAV^* and *Cre*^+^/*Setdb2 ^AAV^* mice (n = 6). Scale bar, 20 μm.

(E-H) Representative images of HE, Masson, α-SMA(IHC), Vimentin (IF) and quantitative analysis in glomeruli from different groups of mice (n = 6). Scale bar, 20 μm.

Data are presented as Mean±SD. Two-tailed Student’s unpaired t-test analysis (B, D), Two-way ANOVA followed by Tukey’s post-test (F-H), with *p* values indicated.


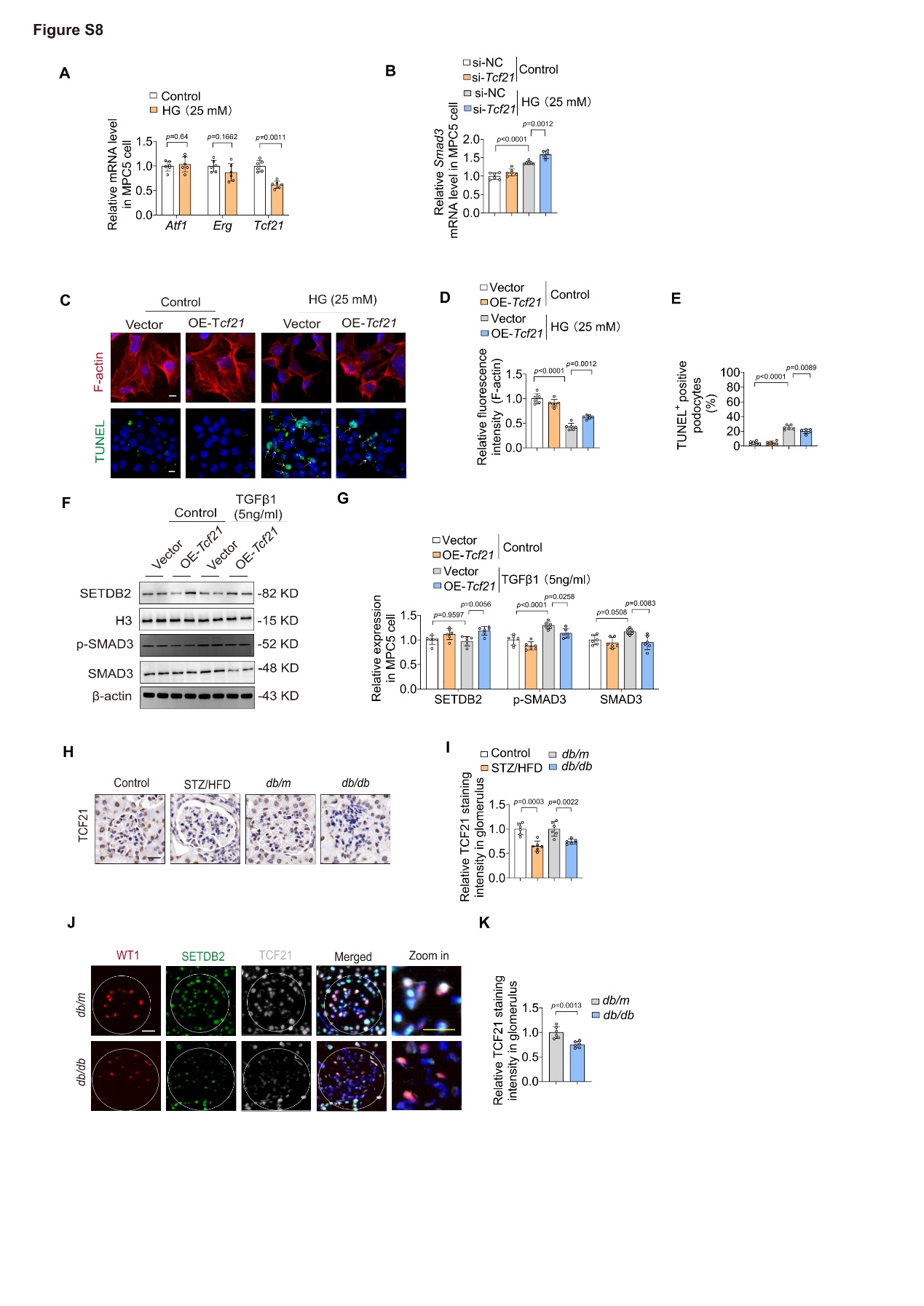


**Figure S8. TCF21 promotes SETDB2 transcription by directly binding to its promoter. Related to Figure 6.**

(A) Relative mRNA level of *Atf1*, *Erg* and *Tcf21* in MPC5 in the presence or absence of HG (n = 6).

(B) Relative mRNA level of *Smad3* in MPC5 transfected with *Tcf21* siRNA in the presence or absence of HG. (n = 6).

(C-E) Representative IF images of F-actin (top, red) and TUNEL (bottom, green) staining and quantitative analysis in MPC5 transfected with *Tcf21* overexpression for 48 hours in the presence or absence of HG (n = 6). Scale bar, 10 μm.

(F-G) Representative Western blot images of SETDB2, p-SMAD3 and SMAD3 expression and quantitative analysis in MPC5 cells with TCF21 overexpression treatments in the presence or absence of TGFβ1 (n = 6).

(H-I) Representative IHC images of TCF21 and quantitative analysis in glomeruli from control and DKD mice (n = 6). Scale bar, 20 μm.

(J-K) Representative confocal microscopic images showing the expression of TCF21 (white), SETDB2 (green) and WT-1 (red) in *db/m* and *db/db* mice (n = 6). Scale bar, 20 μm(white), 20 μm (yellow).

Data are presented as Mean±SD. Two-tailed Student’s unpaired t-test analysis (A, I, K), Two-way ANOVA followed by Tukey’s post-test (B, D, E, G), with *p* values indicated.

**Table S1. Clinical characteristics and relative glomeruli SETDB2 staining levels in renal biopsy.**

| Number | Age (year) | Sex | Body weight (kg) | GLU (mmol/l) | Years of diabetes | SCr (μmol/l) | UPE/24 h (g) | eGFR (ml/min/1.73m2) | Relative SETDB2 staining intensity |
| --- | --- | --- | --- | --- | --- | --- | --- | --- | --- |
| 1 | 42 | Male | 73 | 5.32 | 0 | 60.5 | ＜0.15 | 117.48 | 1.02 |
| 2 | 43 | Male | 81 | 5.22 | 0 | 75.1 | ＜0.15 | 111.77 | 0.97 |
| 3 | 51 | Female | 75 | 5.74 | 0 | 49.3 | ＜0.15 | 108.45 | 0.71 |
| 4 | 56 | Male | 82 | 4.94 | 0 | 72.9 | ＜0.15 | 105.75 | 1.28 |
| 5 | 55 | Female | 60 | 5.08 | 0 | 61.1 | ＜0.15 | 98.26 | 0.85 |
| 6 | 61 | Female | 69 | 5.19 | 0 | 56.4 | ＜0.15 | 96.72 | 0.95 |
| 7 | 63 | Male | 78 | 4.5 | 0 | 75.5 | ＜0.15 | 96.50 | 1.28 |
| 8 | 67 | Female | 73 | 5.94 | 0 | 50.6 | ＜0.15 | 96.10 | 1.16 |
| 9 | 65 | Male | 52 | 5.04 | 0 | 79.5 | ＜0.15 | 95.92 | 0.76 |
| 10 | 53 | Male | 85 | 5.42 | 0 | 78.9 | ＜0.15 | 93.44 | 1.22 |
| 11 | 60 | Male | 79 | 5.9 | 0 | 79.8 | ＜0.15 | 92.17 | 1.12 |
| 12 | 40 | Male | 72 | 5.99 | 0 | 90.5 | ＜0.15 | 91.10 | 0.75 |
| 13 | 40 | Male | 68 | 5.74 | 0 | 90.5 | ＜0.15 | 91.10 | 1.23 |
| 14 | 62 | Female | 68 | 5.44 | 0 | 54 | ＜0.15 | 90.82 | 1.01 |
| 15 | 61 | Female | 49 | 6.09 | 0 | 45.2 | ＜0.15 | 90.39 | 0.70 |
| 16 | 57 | Male | 66 | 4.96 | 10 | 82.9 | 1.59 | 89.89 | 0.79 |
| 17 | 38 | Male | 100 | 12.1 | 10 | 94.1 | 15.96 | 88.14 | 0.78 |
| 18 | 63 | Female | 74 | 5.82 | 13 | 65.7 | 0.43 | 86.04 | 0.98 |
| 19 | 55 | Male | 61 | 6.16 | 5 | 90.2 | 1.8 | 82.32 | 0.78 |
| 20 | 54 | Male | 75 | 4.58 | 9 | 90.9 | 0.1 | 82.13 | 1.13 |
| 21 | 66 | Male | 79 | 6.21 | 10 | 89.7 | 1.1 | 76.72 | 1.00 |
| 22 | 59 | Male | 87 | 7.12 | 8 | 94.2 | 0.8 | 75.95 | 0.87 |
| 23 | 32 | Male | 83 | 5.04 | 8 | 113.6 | 0.13 | 73.21 | 0.52 |
| 24 | 54 | Female | 75 | 6.16 | 10 | 81.7 | 4.12 | 70.43 | 1.06 |
| 25 | 44 | Male | 78 | 7.6 | 15 | 111 | 1.52 | 69.20 | 0.91 |
| 26 | 60 | Female | 62 | 6.63 | 15 | 82.1 | 5.04 | 67.12 | 0.69 |
| 27 | 49 | Female | 60 | 8.93 | 23 | 87.8 | 0.85 | 66.86 | 0.55 |
| 28 | 72 | Male | 53 | 8.13 | 13 | 99.6 | 3.41 | 64.81 | 0.55 |
| 29 | 62 | Female | 75 | 9.92 | 10 | 83.9 | 1.5 | 64.47 | 1.29 |
| 30 | 62 | Female | 70 | 9.64 | 16 | 87.4 | 1.99 | 61.36 | 0.61 |
| 31 | 64 | Male | 67 | 8.68 | 20 | 111.6 | 1.18 | 59.74 | 0.61 |
| 32 | 56 | Male | 87 | 6.89 | 14 | 119.7 | 0.84 | 58.06 | 0.89 |
| 33 | 76 | Female | 62 | 5.69 | 13 | 85.4 | 2.46 | 57.19 | 1.04 |
| 34 | 53 | Male | 94 | 8.13 | 9 | 123.9 | 4.28 | 56.88 | 0.52 |
| 35 | 72 | Male | 59 | 9.24 | 17 | 111.9 | 3.16 | 56.29 | 0.79 |
| 36 | 64 | Male | 78 | 4.88 | 11 | 123.6 | 0.89 | 52.80 | 0.79 |
| 37 | 67 | Female | 80 | 8.41 | 7 | 97.8 | 1.34 | 51.72 | 0.74 |
| 38 | 63 | Male | 87 | 7.15 | 0.75 | 129 | 0.9 | 50.50 | 1.00 |
| 39 | 54 | Female | 56 | 6.22 | 5 | 110.4 | 0.28 | 48.94 | 1.06 |
| 40 | 62 | Male | 60 | 7.41 | 4 | 134.8 | 1.009 | 48.22 | 1.02 |
| 41 | 64 | Female | 64 | 7.34 | 8 | 107.1 | 1.23 | 47.32 | 1.21 |
| 42 | 43 | Male | 78 | 4.79 | 10 | 153 | 0.89 | 47.28 | 0.62 |
| 43 | 66 | Female | 61 | 4.78 | 24 | 106.3 | 1.72 | 47.09 | 1.09 |
| 44 | 49 | Female | 89 | 4.47 | 10 | 118.4 | 1.95 | 46.58 | 0.90 |
| 45 | 41 | Male | 105 | 5.38 | 1 | 159.3 | 1.47 | 45.67 | 0.75 |
| 46 | 43 | Male | 75 | 4.69 | 20 | 163 | 4.53 | 43.80 | 0.98 |
| 47 | 43 | Male | 71 | 7.29 | 0.5 | 163.4 | 2.72 | 43.67 | 0.85 |
| 48 | 55 | Male | 61 | 5.38 | 15 | 155.8 | 0.55 | 42.52 | 0.75 |
| 49 | 65 | Male | 79 | 5.97 | 10 | 147.6 | 4.51 | 42.31 | 0.98 |
| 50 | 71 | Male | 61 | 7.12 | 12 | 152.3 | 1.17 | 39.05 | 0.97 |
| 51 | 49 | Female | 56 | 6.45 | 3 | 137.1 | 0.81 | 39.01 | 0.64 |
| 52 | 55 | Female | 64 | 8.17 | 15 | 134.9 | 3.45 | 38.14 | 0.75 |
| 53 | 60 | Male | 74.5 | 5 | 15 | 294.7 | 1.88 | 18.99 | 0.67 |
| 54 | 70 | Male | 69 | 6.49 | 13 | 159.8 | 4.13 | 37.11 | 0.71 |
| 55 | 56 | Female | 63 | 5.97 | 13 | 141.8 | 2.13 | 35.66 | 0.90 |
| 56 | 45 | Male | 65 | 4.05 | 12 | 192.5 | 0.16 | 35.32 | 1.09 |
| 57 | 61 | Female | 68 | 9.45 | 11 | 139.3 | 2.04 | 35.17 | 0.97 |
| 58 | 74 | Male | 78 | 12.07 | 20 | 213.3 | 3.78 | 25.45 | 0.67 |
| 59 | 63 | Male | 70 | 3.02 | 23 | 242.9 | 6.62 | 23.50 | 0.71 |
| 60 | 52 | Male | 87 | 3.89 | 5 | 304.7 | 2.42 | 19.30 | 0.62 |

**Table S2. Physical and biochemical parameters of STZ/HFD-induced and db/db diabetic mice.**

| Group | Control | STZ/HFD | db/m | db/db |
| --- | --- | --- | --- | --- |
| BW (g) | 28.98±1.37 | 25.42±1.22*** | 32.47±1.95 | 52.17±3.78^####^ |
| FBG (mmol/l) | 6.48±0.73 | 25.62±1.6**** | 5.78±0.81 | 27.07±2.87^####^ |

BW, body weight; FBG, fasting blood glucose; Data are expressed as means ± SD. ** ****p* < 0.001, *****p* < 0.0001 vs. control mice (n = 6) or ^####^*p* < 0.0001 vs. db/m mice (n = 6). Two-tailed Student’s t-test analysis was used.

**Table S3. Physical and biochemical parameters of STZ/HFD-induced diabetic mice.**

| Group | Control | | STZ/HFD | |
| --- | --- | --- | --- | --- |
|  | Cre^-^/Setdb2^fl/fl^ | Cre^+^/Setdb2^fl/fl^ | Cre^-^/Setdb2^fl/fl^ | Cre^+^/Setdb2^fl/fl^ |
| BW (g) | 28.23±1.22 | 29.47±1.73 | 24.72±1.18*** | 23.38±1.73*** |
| FBG (mmol/l) | 5.37±0.53 | 5.4±0.45 | 24.8±3.2**** | 25.73±3.35**** |

BW, body weight; FBG, fasting blood glucose; Data are expressed as means ± SD. ****p* < 0.001, *****p* < 0.0001 vs. Cre^-^/Setdb2^fl/fl^ control mice (n = 6). Two-way ANOVA followed by Tukey’s post-hoc test was used.

**Table S4. Physical and biochemical parameters of STZ/HFD-induced diabetic mice.**

| Group | Control | | STZ/HFD | |
| --- | --- | --- | --- | --- |
|  | Cre^-^/Setdb2^AAV^ | Cre^+^/Setdb2^AAV^ | Cre^-^/Setdb2^AAV^ | Cre^+^/Setdb2^AAV^ |
| BW (g) | 28.62±1.47 | 28.95±1.01 | 24.62±1.09*** | 25.78±1.2**** |
| FBG (mmol/l) | 5.73±0.51 | 5.65±0.59 | 24.7±2.51*** | 22.95±3.07**** |

BW, body weight; FBG, fasting blood glucose; Data are expressed as means ± SD. ****p* < 0.001, *****p* < 0.0001 vs. Cre^-^/Setdb2^AAV^ control mice (n = 6). Two-way ANOVA followed by Tukey’s post-hoc test was used.

**Table S5. Antibodies used in this study.**

| Primary antibodies | Supplier | Product ID | Host | Application |
| --- | --- | --- | --- | --- |
| SETDB2 | Abcam | Ab198817 | Rabbit | WB (1:1000), IHC (1:200) |
| SETDB2 | Abclonal | A7391 | Rabbit | WB (1:500) |
| SETDB2 | Invitrogen | PA5-100857 | Rabbit | IF (1:200),  CUT&Tag (1μl/50μl reaction) |
| Synaptopodin | Servicebio | GB151379 | Mouse | IF (1:200) |
| CD31 | Proteintech | 66065-2-lg | Mouse | IF (1:200) |
| Desmin | Abcam | GB15075 | Rabbit | IF (1:200) |
| α-SMA | Proteintech | 14395-1-AP | Rabbit | IHC (1:200), WB (1:800) |
| WT-1 | Abcam | ab89901 | Rabbit | IF (1:200) |
| Nephrin | Proteintech | 66970-1-Ig | Mouse | IF (1:200) |
| Podocin | Proteintech | 20384-1-AP | Rabbit | IF (1:200), WB (1:1000) |
| H3 | Proteintech | 17168-1-AP | Rabbit | WB (1:2000) |
| H3k9me3 | Abcam | ab176916 | Rabbit | CHIP (4μl/reaction)  CUT&Tag (1μl/50μl reaction) |
| H3k9me3 | Active Motif | ab176916 | Rabbit | WB (1:2000), IHC (1:200) |
| H3k9me2 | Abcam | ab1220 | Mouse | WB (1:1000), IHC (1:200) |
| H3k9me1 | Abcam | ab176880 | Rabbit | WB (1:1000), IHC (1:200) |
| SMAD3 | Proteintech | 66516-1-Ig | Mouse | IF (1:200), IHC (1:200), WB (1:1000) |
| Phospho-Smad3 (Ser425) | Bioss | bs-5616R | Rabbit | WB (1:500) |
| NEDD4L | Proteintech | 13690-1-AP | Rabbit | WB (1:1000) |
| DUSP1 | Proteintech | 67101-1-Ig | Rabbit | WB (1:2000) |
| TβR1 | Abcam | ab235578 | Rabbit | WB (1:1000) |
| Phospho-TβR1 (SerS165) | Abcam | ab112095 | Rabbit | WB (1:1000) |
| E-cadherin | Proteintech | 20874-1-AP | Rabbit | WB (1:1000) |
| Vimentin | Proteintech | 10366-1-AP | Rabbit | IF (1:200), WB (1:1000) |
| Fibronectin | Proteintech | 66042-1-Ig | Mouse | IF (1:200) |
| Bax | Proteintech | 50599-2-Ig | Rabbit | WB (1:800) |
| Bcl2 | Proteintech | 26593-1-AP | Rabbit | WB (1:800) |
| TCF21 | Santa Cruz | sc-377225 | Mouse | IF (1:100), IHC (1:100), WB (1:500) |
| HA | Proteintech | 51064-2-AP | Rabbit | CHIP(4μl/reaction) |
| β-Actin | Proteintech | 66009-1-Ig | Mouse | WB (1:20000) |
| CoraLite488-conjugated Goat Anti-Rabbit IgG(H+L) | Proteintech | SA00013-2 | Goat | IF (1:200) |
| CoraLite594 – conjugated Goat Anti-Rabbit IgG(H+L) | Proteintech | SA00013-4 | Goat | IF (1:200) |
| HRP-conjugated Goat Anti-Rabbit IgG(H+L) | Proteintech | SA00001-2 | Goat | WB (1:2000) |
| HRP-conjugated Goat Anti-Mouse IgG(H+L) | Proteintech | SA00001-1 | Goat | WB (1:2000) |

**Table S6. Primer pairs of target genes used for PCR in this study.**

| **Gene** | **Species** | **Primer sequences** |
| --- | --- | --- |
| *Setdb2*-KO | Mouse | Forward: 5’-AGTTGTGAACTGCCTAACATTAGC-3’  Reverse: 5’-TTTGAAACAGCTTCCCCATGTCG-3’ |
| *Setdb2-Loxp* | Mouse | Forward: 5’-CAAAGCATGTAAGGGGATAT-3’  Reverse: 5’-ACCACAGGCTCATTACAACTGATA-3’ |
| *Podocin-Cre* | Mouse | Forward: 5’-GCGCTGCTGCTCCAG-3’  Reverse: 5’-CGGTTATTCAACTTGCACCA-3’ |
| *Setdb*2-Exon5KO | Mouse | Forward: 5’-GAAGCCACTCTGAGTAACACG-3’  Reverse: 5’-AGAGGGAACCGCACTTGATT-3’ |
| *Setdb*2 | Mouse | Forward: 5’-AATCCTCTGCCAATGTGGGTT-3’  Reverse: 5’-CCCTGAACAAAGGAAACAGAAGT-3’ |
| *Setdb1* | Mouse | Forward: 5’-ACTGAGCAAAGATGGGGACC-3’  Reverse: 5’- ACTCGACTGCCCACAAACAG-3’ |
| *Suv39h1* | Mouse | Forward: 5’-CCTGGACTACGTGGAAGACG-3’  Reverse: 5’-GGGGTAGTCGCTCATCAAGG-3’ |
| *Suv39h2* | Mouse | Forward: 5’-CCAGGCACTCCCATCTACG-3’  Reverse: 5’-TGTCATAGAACTGTCCCCGTC-3’ |
| *Ehmt1* | Mouse | Forward: 5’- GCCTCTCGAGTCAACAATGC-3’  Reverse: 5’- TCACCTCTTTGGCCTTGGAA -3’ |
| *Ehmt2* | Mouse | Forward: 5’- CACTCATCGGGGACGAACC -3’  Reverse: 5’- GGGGAAGAGGGGAACGACTT -3’ |
| *Smad3* | Mouse | Forward: 5’- GTCACTGGATGGTCGGCTG-3’  Reverse: 5’- TGGCCCGTAATTCATGGTGG-3’ |
| *Tgfb1* | Mouse | Forward: 5’- CCGCAACAACGCCATCTATG -3’  Reverse: 5’- CTCTGCACGGGACAGCAAT -3’ |
| *Atf1* | Mouse | Forward: 5’- CTCCTCACAGAAAGCCCACG-3’  Reverse: 5’- ACTGTCCGCTGCTAGTCTGA -3’ |
| *Erg* | Mouse | Forward: 5’- CACCAGTAGTCGCCTTGCTAATC -3’  Reverse: 5’- CGCCGAGCCACCTCATCC -3’ |
| *Tcf21* | Mouse | Forward: 5’- TGACGTGGCCCTTTATGGTG-3’  Reverse: 5’- TAGGGAGAGGAGCGATGCAA-3’ |
| *Smad3-p1* | Mouse | Forward: 5’- CTTCAGGGCTGACTCTGTGG-3’  Reverse: 5’- ACGGATTTGGGGCGTTACAT-3’ |
| *Smad3-p2* | Mouse | Forward: 5’- CAGATGTCTTTGAGGCCCGT-3’  Reverse: 5’-AGTGGCCAGAGCTGCTTTAG -3’ |
| *Smad3-p3* | Mouse | Forward: 5’-CGTGCGGAAACCCAAACTTT -3’  Reverse: 5’-AACTCTGGAGAACTTGCCCG -3’ |
| *Setdb2-p1* | Mouse | Forward: 5’-CTGTGGCAGAAAGATCCGGT -3’  Reverse: 5’-GCCGCCAGCATTCATTCAAT -3’ |
| *Setdb2-p2* | Mouse | Forward: 5’- CCTGATCCGGCTTTCAACCT-3’  Reverse: 5’- AAATGGGCTCCAACTGCAGT-3’ |
| *Setdb2-p3* | Mouse | Forward: 5’- AATCTTCGCGCCACAAACAC-3’  Reverse: 5’-GGTGACAGGAGTGTTGGGAG-3’ |
| *β-actin* | Mouse | Forward: 5’-GGCTGTATTCCCCTCCATCG-3’  Reverse: 5’-CCAGTTGGTAACAATGCCATGT-3’ |

**Table S7. The sequence of siRNA used for target knockout in this study.**

| **Name** | **Species** | **Sequences** |
| --- | --- | --- |
| *Smad3* siRNA | Mouse | sense: 5ʹ-CAGCACACAAUAACUUGGACCUACA-3ʹ  antisense: 5ʹ-AUGUAACCGUUCUCGUACUUGTT-3 |
| *Nr2c2* siRNA | Mouse | sense: 5ʹ-GCAUGGCGAAACUGGAUAUAGTT-3ʹ  antisense: 5ʹ-CUAUAUCCAGUUUCGCCAUGCTT-3 |
| *Atf1* siRNA | Mouse | sense: 5ʹ-GAGUAACACGACAGAGACUGC-3ʹ  antisense: 5ʹ- AGUCUCUGUCGUGUUACUCUU-3 |
| *Runx1t1* siRNA | Mouse | sense: 5ʹ- CAAGCGACCAUGCACUAUUAGTT-3ʹ  antisense: 5ʹ-CUAAUAGUGCAUGGUCGCUUGTT-3 |
| *Ets1* siRNA | Mouse | sense: 5ʹ-CGGUAUCGAGCAUGCUCAGUGUGUU-3ʹ  antisense: 5ʹ-AACACACUGAGCAUGCUCGAUACCG-3 |
| *Klf5* siRNA | Mouse | sense: 5ʹ-GCGAUUCACAACCCAAAUUUATT-3ʹ  antisense: 5ʹ-UAAAUUUGGGUUGUGAAUCGCTT-3 |
| *Tcf7l2* siRNA | Mouse | sense: 5ʹ-GGAAUGCAGUGCCGUUUCUUU-3ʹ  antisense: 5ʹ-AGAAACGGCACUGCAUUCCUU-3 |
| *Gabpa* siRNA | Mouse | sense: 5ʹ-AGCUUAGUGUACAGGUAAUUUTT-3ʹ  antisense: 5ʹ-AAAUUACCUGUACACUAAGCUTT-3 |
| *Tcf12* siRNA | Mouse | sense: 5ʹ-CCAUCCCAUAAUGCAUCAAUUTT-3ʹ  antisense: 5ʹ-AAUUGAUGCAUUAUGGGAUGGTT-3 |
| *Erg* siRNA | Mouse | sense: 5ʹ-AGCGCUACGCCUACAAGUUUGTT-3ʹ  antisense: 5ʹ-CAAACUUGUAGGCGUAGCGCUTT -3 |
| *Tcf21* siRNA | Mouse | sense: 5ʹ-CAAGUACGAGAACGGUUACAUTT-3ʹ  antisense: 5ʹ-AUGUAACCGUUCUCGUACUUGTT-3 |
| *Brd2* siRNA | Mouse | sense: 5ʹ-CCCGGAAGCCCUACACUAUUATT-3ʹ  antisense: 5ʹ-UAAUAGUGUAGGGCUUCCGGGTT-3 |


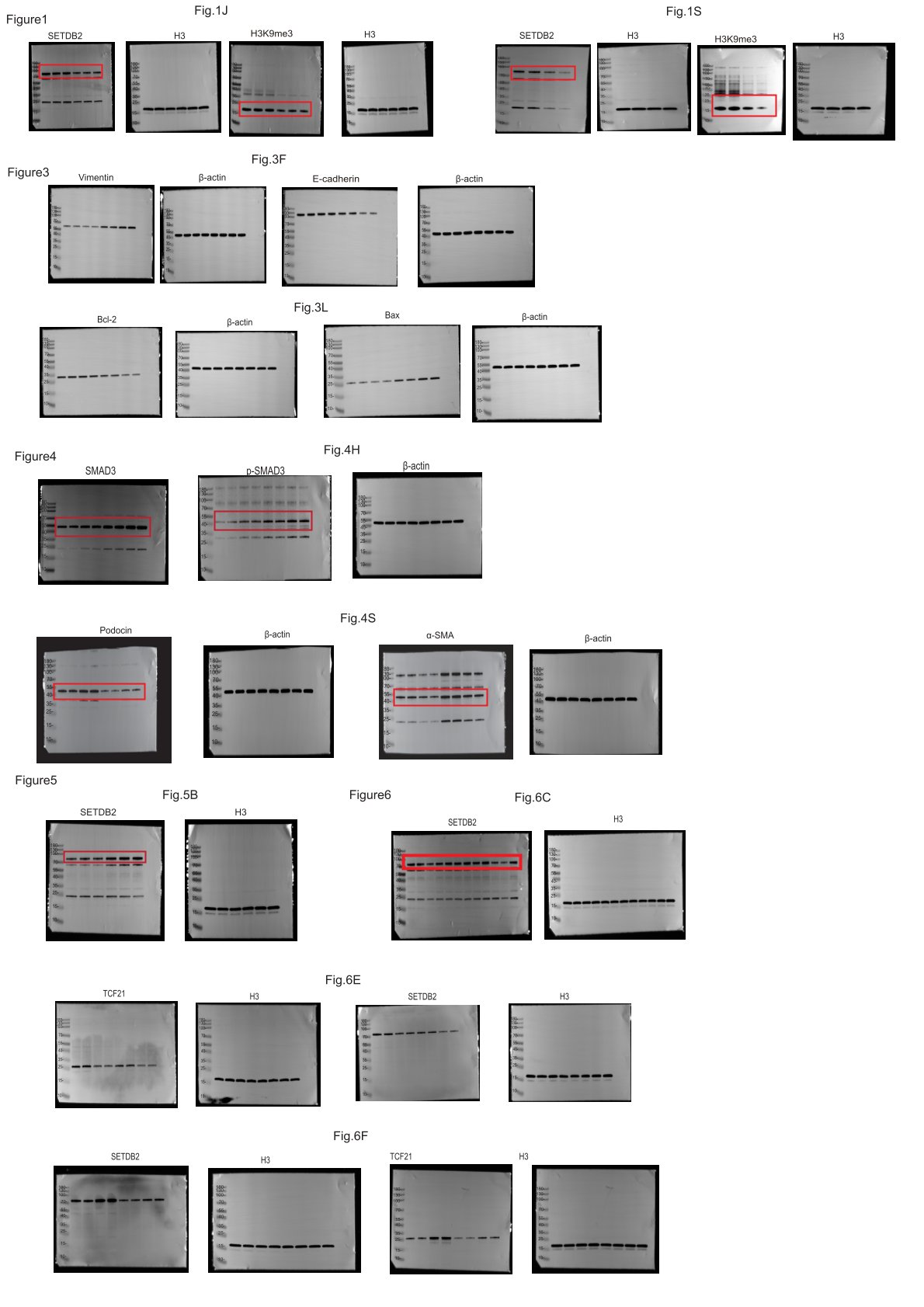


**Figure S9. Full scans of representative immunoblots. Related to Figure 1-6.**


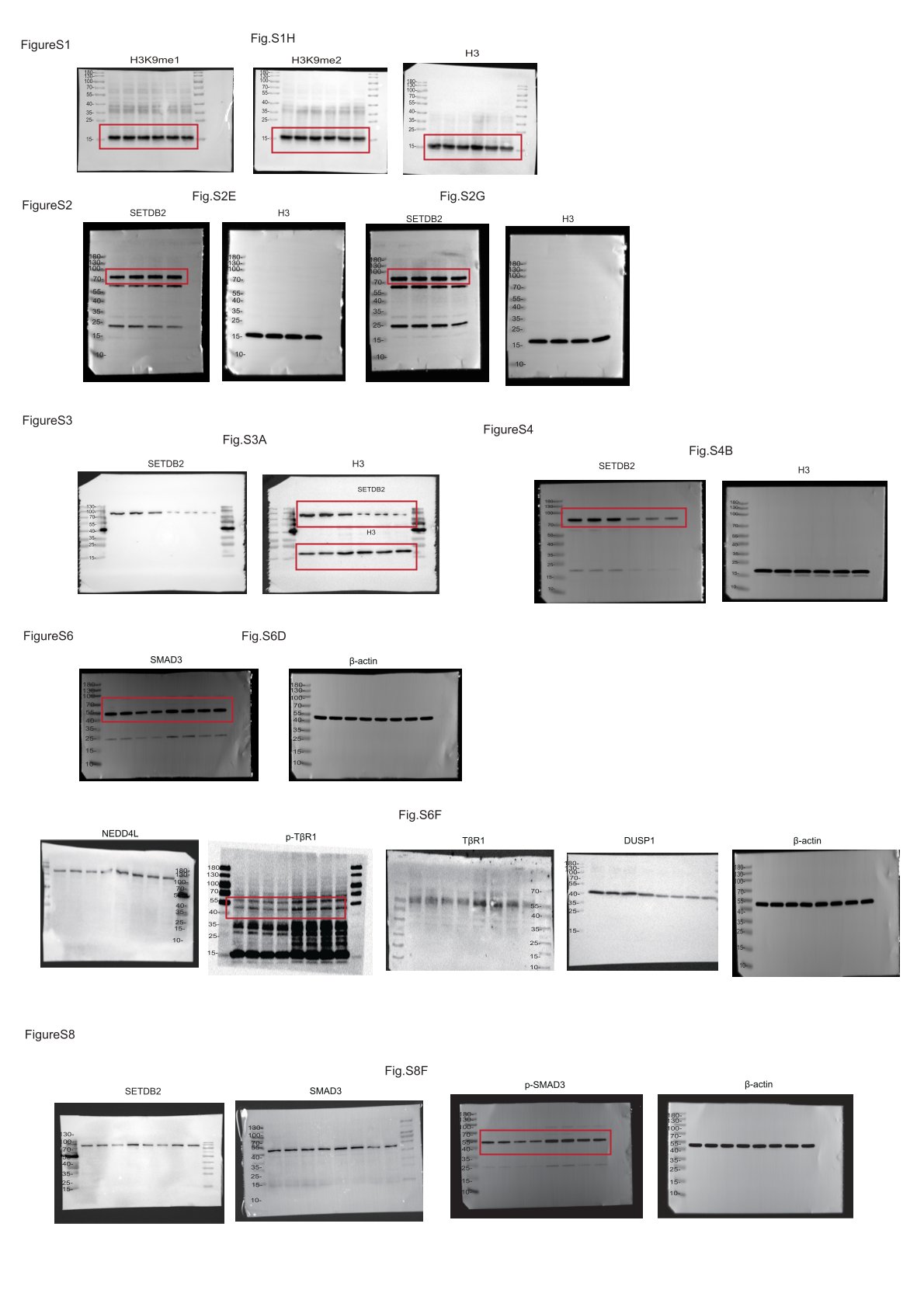


**Figure S10. Full scans of representative immunoblots. Related to Figure S1-8.**

**DETAILED METHODS**

**Human renal biopsy samples**

Renal biopsies were performed as part of routine clinical diagnostic investigation and collected. Sections of renal biopsies were obtained from Nephrology Department of China-Japan Friendship Hospital. A total of 60 renal tissue specimens were collected with the detailed information described in Supplemental Table S1. The 15 control samples(eGFR>90mL/min/1.73 m²) were para-carcinoma tissues from patients which underwent tumor nephrectomy and were not diagnosed with diabetes or other kidney diseases. The 45 DKD samples of renal biopsies diagnosed as DKD were classified in accordance with a pathologic classification provided by the Renal Pathology Society. From mild to severe, DKD was divided into four hierarchical glomerular lesions as follows: mild DKD(eGFR 60~90mL/min/1.73m²); moderate DKD(45~60mL/min/1.73m²); severe DKD(eGFR<45mL/min/1.73m²); The investigations were conducted in accordance with the principles of the Declaration of Helsinki and approved by the Research Ethics Committee of China-Japan Friendship Hospital (Document No. 2024-KY-129-1) after informed consent was obtained from the subjects.

**Animal studies**

Male mice were randomly allotted to the experiments. Mice were kept at 55% humidity and 22 °C with a 12 day-night rhythm for all the experimental conditions. All experimental protocols for animal studies were approved by the Institutional Animal Care and Use Committee of China-Japan Friendship Hospital, and conducted in accordance with the National Institutes of Health Guide for the Care and Use of Laboratory Animals (Document No. ZRDWLL240126).

**Generation of Global *Setdb2* knockout mice:** Global Setdb2 knockout homozygous mice were obtained by self-crossing of knockout Setdb2 heterozygous mice (C57BL/6JCya-*Setdb2^em1^*/Cya, S-KO-07036, Cyagen, China). In these mice, Setdb2 exon 3-7 which covers 46.25% of the coding region was deleted, resulting in loss of the signal peptide and disrupts its open reading frame (ORF). Genotyping by tail preparation and PCR (Table S6) were performed at 2 weeks of age. Wide type (*Setdb2*^+/+^) yields only a 606 bp band; homozygous (*Setdb2*^-/-^) yields only a 711 bp band.

**Generation of podocyte-specific *Setdb2* knockout mice:** Floxed *Setdb2* mice (C57BL/6JCya-*Setdb2^em1flox^*/Cya, S-CKO-08090, Cyagen, China) were crossed with mice expressing Cre recombinase (Cre) under the control of the podocin promoter (Nphs2-cre; C001027, Cyagen, China) to generate podocyte-specific Setdb2 knockout mice (Nphs2-Cre / *Setdb2*^fl/fl^ mice; *Cre*^+^ / *Setdb2*^fl/fl^ mice). Mice with two flox alleles and without Cre expression were used as controls (*Setdb2*^fl/fl^ mice; Cre^-^/*Setdb2*^fl/fl^ mice). Genotyping by tail preparation and PCR([Table S6](https://www.sciencedirect.com/science/article/pii/S1074761323004995?via%3Dihub#mmc1)) were performed at 2 weeks of age. Wide type (*Setdb2*^+/+^) yields only a 314 bp band; homozygous (*Setdb2*^fl/fl^) yields only a 383 bp band; heterozygous (*Setdb2*^fl/+^) yields both bands. Cre positive (Cre^+^) yields a 200 bp band, but Cre negative (Cre^-^) has no band.

**Streptozotocin/ High-fat diet (STZ/HFD) combined uninephrectomy treatment -Induced Diabetic Kidney Disease (DKD) in Mice**

To induce DKD in C57BL/6 mice, a low dose of streptozotocin (STZ, S0130, Sigma) and high-fat diet (STZ/HFD) was administered along with uninephrectomy treatment according to established protocols. Four-week-old male mice and their littermate control mice were fed either a high fat diet (HFD, 60 kcal% fat, 20% carbohydrate and 20% protein, Research Diets, D12492) or control diet (NCD, Research Diets, D12450J) for 4 weeks and then uninephrectomized. After one week recovery period from uninephrectomy, mice were injected low-dose STZ dissolved in 8 mg/ml sodium citrate buffer (pH 4.5) (40 mg/kg body weight intraperitoneally daily for 5 days) after starving for 8 h to induce partial insulin deficiency or injected vehicle for control. Mice continued to be maintained on a HFD or NCD for another 15 weeks (as shown in Figure 2). All the mice had free access to food and water, and their body weight and fasting blood glucose levels were measured at the twenty-four-week-old (TableS2-4). Simultaneously, 24 h urine samples were collected for biochemical analysis, after which the mice were euthanized and kidney tissues were harvested for further examination.

**Spontaneous type 2 diabetic *db/db* mice**

Heterozygote BKS *db/m* mice and homozygote BKS *db/db* mice were purchased from Cyagen Biosciences, in the China. The *db/db* mice produced identifiable obesity phenotypes at 3–4 weeks of age, with elevated blood glucose at 4–8 weeks. The db/m mice were used as genetic control. Proteinuria will be observed at the age of 10–20 weeks as a marker of successful establishment of type 2 DKD models.

**Adriamycin (ADR)-induced Nephropathy in Mice**

In the ADR model, male mice (7 weeks of age) were administered ADR (D409016, Aladdin, Shanghai, 20 mg/kg) intravenously by tail vein injection. Urine was collected weekly to assess for albuminuria, and mice were sacrificed at 9 weeks after proteinuria. At the end of the study, blood and 24 h urine samples were collected for biochemical testing. Simultaneously, the mice were euthanized and the kidney tissue samples were harvested for histopathological analysis.

**Podocyte-specific *Setdb2* overexpression in Mice**

The CDS region of *Setdb2* was inserted into the polyclonal site of the pHBAAV-CMV-DIO-MCS vector to generate the adeno-associated virus pHBAAV-CMV-DIO-MCS-*Setdb2*(Hanheng, China). The pHBAAV-CMV-DIO-MCS vector was synthesized without any modification as a control group. Podocyte-specific *Setdb2* overexpression mice were generated by tail vein injecting adeno-associated virus into Nphs2-Cre mice, with a dose of 1×10^8^ genome copies per mouse. The elevated SETDB2 expression in podocyte will be observed at 8-10 weeks after injecting as a marker of successful establishment of podocyte-specific *Setdb2* overexpression mice.

**Isolation of glomeruli**

Glomeruli were isolated using a modified method described in previous studies^[1]^. In brief, mice were anesthetized and the surgical procedures were performed as follows: 1) the distal abdominal aorta, the distal inferior cava vein were ligated; 2) the superior mesenteric and celiac arteries were ligated; 3) the proximal abdominal aorta was ligated above the celiac arteries; 4) the abdominal aorta together with inferior cava vein were clipped with vessel clamps below the renal artery and vein; 5) an intravenous infusion needle was inserted into the middle of the abdominal aorta and fixed in place; 6) the vessel clamp was removed; 7) a small hole was cut in the inferior cava vein to give an outlet in order to make a complete circuit. After rinsing the remaining blood in vessels with ice-cold sterile Hanks’ balanced salt solution (HBSS), kidneys were perfused slowly with inactivated Dynabeads (8 ×10^7^ beads/mouse, Invitrogen, M-450 Tosylactivated) diluted in 20 mL prewarmed (37 ℃) HBSS through the intravenous infusion needle. Kidneys were removed, minced into small pieces in pre-cooled tubes and digested with buffer containing collagenase II (C6885, Sigma) at 37℃ for 30 min with gentle agitation. The digested tissue was then gently pressed through a 100 µm cell strainer on ice, followed by ice-cold HBSS flushing. The cell suspension was centrifuged at 200g at 4℃ for 5 min. Finally, the pellet was resuspended and glomeruli containing Dynabeads were gathered by a magnetic particle concentrator after at least three times washing with ice-cold HBSS. Glomeruli purity was determined by inspecting 20 μL aliquots of glomeruli suspensions on glass slides using a light microscope. Lastly, the extracted glomeruli were stored at −80 ℃ until further examination.

**Mouse primary podocytes isolation and culture**

Mouse primary podocytes were isolated as previously described. Mouse kidneys were cut into small pieces and digested with buffer containing collagenase II at 37 ℃ for 30 min with gentle agitation. The small kidney fragments were sieved through 100 μm metal sieve with drop wise addition of HBSS. The filtrate was collected and passed through 40 μm sieves. The glomeruli were collected on the 40 μm and washed thrice using HBSS. The washed glomeruli were collected in tubes and centrifuged at 300 x g for 5 min. The collected glomeruli were cultured in complete cell culture media (RPMI 1640, 10% FBS) and were differentiated for 10 days for further analysis. Differentiation of the podocytes were confirmed by expression of Nephrin, Synaptopodin and Podocin.

**Cell lines culture and treatments:** Mouse podocytes line 5(MPC5, Cyagen, China), mouse glomerular endothelial cells (GECs, Procell, China), and mouse glomerular mesangial cells (MSCs, Procell, China) were cultured in RPMI 1640 supplemented with 10% fetal bovine serum, 100 U/mL penicillin, and 100 U/mL streptomycin. The cells were treated with high glucose (HG, a final concentration of 12.5 or 25 mmol/L in culture medium) for 48 h.

**Establishment of knockout *Setdb2* MPC5 cell line**

MPC5 cell line in which *Setdb2* were deleted were generated using the CRISPR/Cas9 gene-editing technology mediated by electroporation (Cyagen, China). After electroporation, single colonies were picked and verified by PCR and sequencing, and homozygous cells with the mouse *Setdb2* gene knocked out were successfully obtained.

**RNA sequencing analysis**

Total RNA was extracted using the TRIzol reagent (Invitrogen, CA, USA) according to the manufacturer’s protocol. RNA purity and quantification were evaluated using the NanoDrop 2000 spectrophotometer (Thermo Scientific, USA). RNA integrity was assessed using the Agilent 2100 Bioanalyzer (Agilent Technologies, Santa Clara, CA, USA). Then the libraries were constructed using VAHTS Universal V10 RNA-seq Library Prep Kit (Premixed Version) according to the manufacturer’s instructions. The transcriptome sequencing and analysis were conducted by OE Biotech Co., Ltd. (Shanghai, China). The libraries were sequenced on an Illumina Novaseq 6000 platform and 150 bp paired-end reads were generated. Then clean reads for each sample were retained for subsequent analyses. The clean reads were mapped to the reference genome using HISAT2. FPKM of each gene was calculated and the read counts of each gene were obtained by HTSeq-count. PCA analysis were performed using R (v 3.2.0) to evaluate the biological duplication of samples. Differential expression analysis was performed using the DESeq2. Based on the hypergeometric distribution, GO analysis of DEGs were performed to screen the significant enriched term using R (v 3.2.0), respectively. R (v 3.2.0) was used to draw the column diagram, the chord diagram and bubble diagram of the significant enrichment term. Gene Set Enrichment Analysis (GSEA) was performed using GSEA software. The analysis was used a predefined gene set, and the genes were ranked according to the degree of differential expression in the two types of samples. Then it is tested whether the predefined gene set was enriched at the top or bottom of the ranking list.

**Histological analysis of** **mouse renal tissues**

Mouse renal tissues were fixed with 4% paraformaldehyde (PFA) for 24 h, and then embedded in paraffin. 4 μm transverse sections were cut for pathological analysis under the blind method. Hematoxylin and eosin (H&E), Periodic Acid-Schiff (PAS), and Masson staining were performed according to standard histological protocols. The optical microscope (Nikon Eclipse E100, Japan) and imaging system (Nikon DS-U3, Japan) were used to photograph at 100× magnification. The positive area of the glomeruli was quantified using ImageJ software.

**Urine albumin and creatinine measurements**

Urine creatinine levels were measured using an auto-chemistry analyzer according to the creatinine assay kit. Urine albumin was detected using an ELISA kit (Enzyme-linked Biotechnology, Shanghai, China; Cat No. ml1037585V) according to the manufacturer’s instructions. The urine albumin excretion rate was expressed as the ratio of albumin to creatinine.

**Immunofluorescence staining**

Paraffin‐embedded kidney sections (4 μm) were deparaffinized and antigen retrieval, which was performed under high pressure in citrate buffer for 10 min. In vitro, the cell-climbing pieces were fixed with 4% PFA at 4 °C for 30 min. The permeabilization was performed by 0.5% Triton X-100 for 20 min, and 1% BSA was added to block the cells for 1 hours at room temperature. Then, the sections or cell-climbing pieces were incubated with different primary antibodies at 4 °C overnight, followed by secondary antibodies incubated for 2 h away from light. Nuclei were stained with 4′,6-diamidino-2-phenylindole (DAPI). The details of the antibodies used are listed in Table S2. Fluorescence microscope (Nikon Eclipse C1, Japan) and imaging system (3DHISTECH, Pannoramic MIDI, Hungary) were used to photograph at 50× or 100× magnification. The positive signal was quantified using ImageJ software.

**Immunohistochemistry staining**

The 4 μm sections of paraffin-embedded kidney were deparaffinized, rehydrated, and antigen-retrieved. Slides were incubated in 3% hydrogen peroxide for 15 min, and 1% BSA was added to block the cells for 1 hours at room temperature. Then treated with indicated primary antibodies (listed in Table S5), at 4°C overnight, followed by their corresponding peroxidase-conjugated secondary antibody for 30 min at room temperature. Sections were stained with a DAB kit. Finally, the nuclei were stained with hematoxylin and dehydrated before mounting the slide. The optical microscope (Nikon Eclipse E100, Japan) and imaging system (Nikon DS-U3, Japan) were used to photograph at 100× magnification, and the positive area of the glomeruli was quantified using ImageJ software.

**Transmission electron microscopy (TEM)**

The renal cortex was fixed with a solution containing 3% glutaraldehyde plus 2% paraformaldehyde in 0.1M cacodylate buffer (pH 7.3) overnight at 4°C, and post-fixed with 1% buffered osmium. Cultured podocytes were fixed with a solution including 0.5% glutaraldehyde plus 2% paraformaldehyde. After dehydration and infiltration embedding, the samples were polymerized in a 60 °C oven for approximately 72 h. Ultrathin sections were cut in a Leica Ultracut microtome (Leica), stained with uranyl acetate and lead citrate, then examined and photographed in a HT7800 transmission electronmicroscope (Hitachi).

**Scanning electron microscopy (SEM)**

The renal cortex was fixed with a solution containing 3% glutaraldehyde plus 2% paraformaldehyde in 0.1M cacodylate buffer (pH 7.3) overnight at 4°C, and post-fixed with 1% buffered osmium. The samples were dehydrated with increasing concentrations of ethanol, then transferred to increasing concentrations of hexamethyldisilazane 6 and air dried overnight. Place the sample firmly on the double-sided adhesive of the conductive carbon film and then put it on the sample stage of the MC1000 ion sputtering instrument (Hitachi) for gold spraying for about 30 seconds. Observe the element analysis of the tested area under a scanning SU8100 electron microscope (Hitachi).

**TUNEL Assay**

Cell apoptosis in the kidney section and cells was detected by TUNEL assay following the manufacturer’s protocol (C1088, Beyotime, China). In brief, paraffin‐embedded kidney sections (4 μm) were deparaffinized. In vitro, the cell-climbing pieces were fixed with 4% PFA at 4 °C for 30 min. The protease K was used to retrieve at 37 °C for 15-20 min. Mix TDT enzyme, dUTP and buffer in a ratio of 2:5:50, and add it to the circle to cover the tissue, After incubating at 37 °C for 1h, the cells were stained with DAPI to label the nuclei, Fluorescence microscope (Nikon Eclipse C1, Japan) and imaging system (3DHISTECH, Pannoramic MIDI, Hungary) were used to photograph at 50× or 100× magnification. The positive signal was quantified using ImageJ software.

**F-actin staining**

Paraffin‐embedded kidney sections (4 μm) were deparaffinized and antigen retrieval, which was performed under high pressure in citrate buffer for 10 min. In vitro, the cell-climbing pieces were fixed with 4% PFA at 4 °C for 30 min. The permeabilization was performed by 0.5% Triton X-100 for 20 min, and 1% BSA was added to block the cells for 1 hours at room temperature. Add the fluorescently labeled phalloides peptide (Thermo Fisher Scientific, A12381CN) to the sample, and incubate in the dark at room temperature for 60 min. Nuclei were stained with DAPI. Fluorescence microscope (Nikon Eclipse C1, Japan) and imaging system (3DHISTECH, Pannoramic MIDI, Hungary) were used to photograph at 50× or 300× magnification. The fluorescence intensity was quantified using ImageJ software.

**RNA extraction and real-time RT-PCR**

The TRIzol reagent was used to isolate total RNA according to the manufacturer’s instructions. 1 μg of total RNA was reverse transcribed using a cDNA synthesis kit (Takara, China). The mRNA expression levels were detected by qPCR, which was performed using TB Green PCR Master Mix and a Real-Time PCR System (Takara, China). Primers used are listed in Table S6.

**Western blotting**

Total tissue and cell proteins were extracted from RIPA buffer containing a protease inhibitor cocktail and phosphatase inhibitor. Proteins were separated by 10% sodium dodecyl sulfate-polyacrylamide gel electrophoresis and transferred onto polyvinylidene difluoride (PVDF) membranes. The PVDF membrane was blocked in 5% milk for one hour and incubated with primary antibody at 4°C overnight. The secondary antibody used was horseradish peroxidase (HRP)-labeled goat anti-rabbit/mouse IgG. The details of the antibodies used are listed in Table S5. The blots were visualized using a Bio-Rad imaging system (USA) and quantified using ImageJ software.

**ELISA detection of TGF-β1 in podocytes**

The concentration of TGF-β1 in podocyte culture supernatants was measured using a commercial ELISA kit (NeoBioscience, Cat# EMC107b) according to the manufacturer’s instructions. Podocytes were cultured under normal-glucose (5.5 mmol/L) or high-glucose (25 mmol/L) conditions for 48 h, and the culture media were collected and centrifuged at 3,000 × g for 10 min at 4 °C to remove debris. Total TGF-β1 was determined following the kit protocol. Absorbance was measured at 450 nm using a microplate reader, and concentrations were calculated from the standard curve. Results were expressed as pg/mL and normalized to total protein content.

**siRNA-mediated knockdown and DNA transfections**

Cells were cultured in a medium without antibiotics. Short interfering RNA (siRNA) for target genes or equivalent scramble control (or negative control siRNA), empty vector control plasmid, or plasmid containing target genes were delivered into cells by the Lipofectamine 3000 reagent following the manufacturer's protocol.

**Mitochondrial morphological measurement**

Mitochondrial morphological analysis was conducted as previously described^[2, 3]^. In brief, the mitochondrial aspect ratio was calculated as the ratio of the major to minor axis of each mitochondrion. Mitochondrial cristae were evaluated using a semi-quantitative scoring system (0-4 scale). A score of 0 indicates the complete absence of cristae, 1 represents sparse residual cristae (<25% of normal), 2 denotes partial preservation of cristae (25-50%), 3 indicates largely preserved cristae (50-75%), and 4 corresponds to intact and well-organized cristae (>75% of normal).

**Measurements of oxygen consumption rate (OCR)**

The OCR was measured by Seahorse Bioscience XFe24 Extracellular Flux Analyzers according to the manufacturer’s instructions (Seahorse Bioscience, USA). The data was automatically calculated, recorded, and plotted using XF24 software version 1.8 (Seahorse Bioscience).

**CUT & Tag assay**

CUT&Tag assay was performed using HyperactiveTM In-Situ ChIP Library Prep Kit for Illumina (cat.no.TD903-TD904, Vazyme Biotech, China) according to manufacturer's instruction^[4]^ at Oebiotech Technology Co., Ltd (Shanghai, China). Briefly, prepared concanavalin A-coated magnetic beads (ConA beads) were added to resuspended cells and incubated at room temperature to bound cells. Non-ionic detergent Digitonin was used to permeate cell membrane. Then, primary antibody (H3K9me3 and SETDB2), secondary antibody and the Hyperactive pA-Tn5 Transposase were incubated with the cells that were bounded by ConA beads in order. Therefore, the Hyperactive pA-Tn5 Transposase can exactly cut off the DNA fragments that were bound with target protein. In addition, the cut DNA fragments can be ligated with P5 and P7 adaptors by Tn5 transposase and the libraries were amplified by PCR with the P5 and P7 primers. The purified PCR products were evaluated using the Agilent 2100 Bioanalyzer (Agilent Technologies, Santa Clara, CA, USA). Finally, these libraries were sequenced on the Illumina NovaSeq6000 platform and 150bp paired-end reads were generated for the following analysis.

**CUT & Tag data sequence analysis**

The raw sequence data were firstly quality trimmed by fats software to obtain the clean reads. Then the clean reads were aligned to the reference genome using Bowtie2 and subsequently analyzed by the SEACR software based the stringent parameter to detect genomic regions enriched for multiple overlapping DNA fragments (peaks) that we considered to be putative binding sites. Visualization of peak distribution along genomic regions of interested genes was performed with IGV. Peaks were then annotated using chip-seeker software to obtain the genes and gene annotations about peaks. Significant Motif of peaks were analyzed by MEME and DREME software and aligned to the motif database.

**Chromatin immunoprecipitation (ChIP)**

To investigate the interaction between H3K9me3 and the promoter region of *Smad3* and the interaction between TCF21 and the promoter region of *Setdb2*, a Beyo-ChIPTM Enzymatic ChIP Assay Kit (P2080S, Beyotime, China) was used according to the manufacturer’s procedure in our study. Briefly, cells were crossed link with 1% formaldehyde for 10 min, followed by incubating with SDS lysis buffer containing protease inhibitors. Ultrasound treatment is used to shear the genomic DNA, causing most of the DNA to break down into fragments ranging from 200 to 1000 base pairs in size. After that, 5 ug of specific antibody or normal IgG was added into tubes containing cells lysates and incubated at 4 °C overnight, after which protein G magnetic beads were added incubating at 4 °C for 120 min so that the DNA-protein-antibody complexes could be immobilized on the beads. After the beads were washed, DNA fragments were purified, which was then subjected to q-PCR to quantify the enrichment. The obtained products underwent RT-PCR. The gene-specific primers used are listed in Table S6.

**Scanning of *Setdb2* promoter-binding transcription factors**

Based on either strand of *Setdb2* promoter sequences, multiple prediction databases (TFtarget, TFDB, and GeneCards) was used for scanning the transcription factors on *Setdb2* promoter. The 16 candidate TFs potentially regulating *Setdb2* and 11 transcription factors were consistent with the reduction of *Setdb2* in DKD mice sample.

**JASPAR predicted transcription factors binding sites**

Genomic sequences spanning 2000 bp upstream of the transcription start site were analyzed for relevant transcription factors. Scanning was performed with FIMO (MEME Suite v5.5.3) using a stringent p-value cutoff of 1×10⁻⁵ and relative score threshold >85%. Predicted sites were annotated using ChIPseeker (v1.38.0) and visualized with the UCSC Genome Browser.

**Luciferase reporter gene experiment**

After the plasmid was transfected into the cells for 48 hours, cells were harvested and determined by dual-luciferase reporter assay kit (RG029S, Beyotime, China) according to the manufacturer’s instructions. Briefly, after lysing the reporter gene cells, the luciferase detection reagent of firefly luciferase is added to the sample and the relative light unit (RLU) is detected using a chemiluminescence instrument. Under the condition that the Renilla luciferase is used as the internal reference, the RLU value obtained by the firefly luciferase assay is divided by the RLU value obtained by the Renilla luciferase assay. Based on the obtained ratio, the activation degree of the target reporter gene in different samples is compared.

**References**

1. Fu, Y., et al., *Elevation of JAML Promotes Diabetic Kidney Disease by Modulating Podocyte Lipid Metabolism.* Cell Metab, 2020. **32**(6): p. 1052-1062.e810.1016/j.cmet.2020.10.019.

2. Mise, K., et al., *NDUFS4 regulates cristae remodeling in diabetic kidney disease.* Nat Commun, 2024. **15**(1): p. 196510.1038/s41467-024-46366-w.

3. Lam, J., et al., *A Universal Approach to Analyzing Transmission Electron Microscopy with ImageJ.* Cells, 2021. **10**(9)10.3390/cells10092177.

4. Kaya-Okur, H.S., et al., *CUT&Tag for efficient epigenomic profiling of small samples and single cells.* Nat Commun, 2019. **10**(1): p. 193010.1038/s41467-019-09982-5.
